# Artesunate-induced mitophagy alters cellular redox status

**DOI:** 10.1016/j.redox.2018.07.025

**Published:** 2018-08-04

**Authors:** Jianbin Zhang, Xin Sun, Liming Wang, Yin Kwan Wong, Yew Mun Lee, Chao Zhou, Guoqing Wu, Tongwei Zhao, Liu Yang, Liqin Lu, Jianing Zhong, Dongsheng Huang, Jigang Wang

**Affiliations:** a Department of Oncology, Zhejiang Provincial People's Hospital, People's Hospital of Hangzhou Medical College, Hangzhou 310014, China; b Clinical Research Institute, Key Laboratory of Tumor Molecular Diagnosis and Individual Medicine of Zhejiang Province, Zhejiang Provincial People's Hospital, People's Hospital of Hangzhou Medical College, Hangzhou 310014, China; c Department of Physiology, Yong Loo Lin School of Medicine, National University of Singapore, 117593, Singapore; d Artemisinin Research Center, China Academy of Chinese Medical Sciences, Beijing 100700, China; e Institute of Chinese Materia Medica, China Academy of Chinese Medical Sciences, Beijing 100700, China; f Department of Pharmacology, National University of Singapore, 117600, Singapore; g Key Laboratory of Cardio-cerebrovascular disease prevention & therapy, Gannan Medical University, Ganzhou 341000, China

**Keywords:** ACADVL, very long-chain specific acyl-CoA dehydrogenase, ACC, acetyl-CoA carboxylase 1, ATP, adenosine triphosphate, vacuolar-type H^+^-ATPase (V-ATPase), COX Ⅳ, cytochrome c oxidase IV, CPS, carbamoyl-phosphate synthase, CQ, chloroquine, GSH, glutathione, HSP60, heat shock protein 60 kDa, LAMP1, lysosomal-associated membrane protein 1, LC3, microtubule-associated proteins 1A/1B light chain 3, PINK1, PTEN induced putative kinase 1, PC, pyruvate carboxylase, TOMM20, translocase of outer mitochondrial membrane 20, ROS, reactive oxygen species, SQSTM1/P62, sequestosome 1, TIM23, translocase of the inner membrane 23, VDAC1, voltage-dependent anion-selective channel protein 1, Artesunate, Artemisinin, Mitophagy, PINK1, Parkin, ROS

## Abstract

Artesunate (ART) is a prominent anti-malarial with significant anti-cancer properties. Our previous studies showed that ART enhances lysosomal function and ferritin degradation, which was necessary for its anti-cancer properties. ART targeting to mitochondria also significantly improved its efficacy, but the effect of ART on mitophagy, an important cellular pathway that facilitates the removal of damaged mitochondria, remains unknown. Here, we first observed that ART mainly localizes in the mitochondria and its probe labeling revealed that it binds to a large number of mitochondrial proteins and causes mitochondrial fission. Second, we found that ART treatment leads to autophagy induction and the decrease of mitochondrial proteins. When autophagy is inhibited, the decrease of mitochondrial proteins could be reversed, indicating that the degradation of mitochondrial proteins is through mitophagy. Third, our results showed that ART treatment stabilizes the full-length form of PTEN induced putative kinase 1 (PINK1) on the mitochondria and activates the PINK1-dependent pathway. This in turn leads to the recruitment of Parkin, sequestosome 1 (SQSTM1), ubiquitin and microtubule-associated proteins 1A/1B light chain 3 (LC3) to the mitochondria and culminates in mitophagy. When PINK1 is knocked down, ART-induced mitophagy is markedly suppressed. Finally, we investigated the effect of mitophagy by ART on mitochondrial functions and found that knockdown of PINK1 alters the cellular redox status in ART-treated cells, which is accompanied with a significant decrease in glutathione (GSH) and increase in mitochondrial reactive oxidative species (mROS) and cellular lactate levels. Additionally, knockdown of PINK1 leads to a significant increase of mitochondrial depolarization and more cell apoptosis by ART, suggesting that mitophagy protects from ART-induced cell death. Taken together, our findings reveal the molecular mechanism that ART induces cytoprotective mitophagy through the PINK1-dependent pathway, suggesting that mitophagy inhibition could enhance the anti-cancer activity of ART.

## Highlights

• ART binds to many mitochondrial proteins and causes mitochondrial fission.
• ART treatment leads to autophagy induction and the decrease of mitochondrial proteins.
• When PINK1is knocked down, ART-induced mitophagy is markedly suppressed.
• Knockdown of PINK1 alters the cellular redox status in ART-treated cells.
• ART induces mitophagy through the PINK1-dependent pathway and mitophagy.

## 1 Introduction

Mitophagy is an important cellular pathway that facilitates the removal of damaged mitochondria [1]. Dysregulation of mitochondrial activity results in either programmed cell death or the gross production of reactive oxygen species (ROS) as by-products, which can in turn damage cellular DNA and proteins [2]. During mitophagy, an isolation membrane encircles damaged mitochondria, forming double-membrane autophagosomes, which then undergo fusion with lysosomes for bulk degradation and recycling as nutrients [3]. Thus, mitophagy plays an important role in mitochondrial quality control and is essential for normal cellular functions while being causally related to neurodegenerative and other metabolic diseases [4], [5], [6], such as Parkinson's disease, cardiac defects and even cancer. Many evidences indicate that dysfunction of mitophagy is associated with tumorigenesis [7], [8]. In the absence of the mitophagy machinery, damaged mitochondria produces cytotoxic ROS, which further damage genomic DNA and potentially promote oncogenic mutations [9]. Such genomic instability could eventually lead to the malignant transformation of cells.

The PINK1-Parkin pathway is the most well-described principal mechanism of mitophagy, involving PTEN-induced kinase 1 (PINK1) which contains a mitochondrial targeting sequence, and Parkin, a E3 ligase. Healthy mitochondria maintains a membrane potential that can be used to import PINK1 into the inner membrane where it is cleaved by PARL and cleared from the outer membrane [10]. When mitochondria is damaged or depolarized using mitochondrial uncoupling reagents, such as carbonyl cyanide m-chlorophenylhydrazone (CCCP), mitochondrial uptake and processing of PINK1 is prevented. The resulting accumulation of unprocessed PINK1 on the outer mitochondrial membrane phosphorylates ubiquitin to activate Parkin [11]. Parkin, a RING domain-containing E3 ubiquitin ligase, builds ubiquitin chains on the mitochondrial outer membrane proteins, where they act to recruit autophagy receptors SQSTM1 and other molecules that interact with LC3 for autophagosome formation [12].

Artesunate (ART), a water-soluble derivative of artemisinin, has been widely used for the treatment of malaria [13]. ART is a prodrug that is rapidly converted to its active form dihydroartemisinin (DHA) under biological conditions [14]. Artemisinin-based combination therapy is the recommended first line treatment for *Plasmodium falciparum*-induced malaria and remains the most prominent anti-malarial in use today. Beyond the well-established anti-malarial properties, there is accumulating evidence demonstrating that artemisinin and its derivatives possess cytotoxic effects against many human cancer cell types both in vitro and in vivo [15], [16], [17], [18], [19]. Given the remarkable efficacy and safety of the artemisinin in its anti-malarial role, the potential repurposing of artemisinin into an anti-cancer drug is of great interest. Mechanistically, the cytotoxic effect of artemisinin is believed to be mediated by free iron. The endoperoxide bridge, the pharmacologically active moiety of artemisinin derivatives, is understood to be cleaved in the presence of ferrous iron generating ROS [14]. On the other hand, our previous study [20], [21] revealed that heme, rather than free ferrous iron, is predominantly responsible for artemisinin activation in both parasite cells and cancer cells. Focusing on its anti-cancer properties, we further observed that under treatment with artemisinin in combination with aminolevulinic acid (ALA, a clinically used heme synthesis precursor), the anti-colorectal cancer activity of artemisinin could be greatly enhanced in cell lines as well as in mouse models [21]. In addition, when artemisinin was specifically directed to mitochondria (the site of cellular heme synthesis) using the mitochondria-targeting probe ART-triphenylphosphoniumbromide (ART-TPP) [21], [22], the anti-cancer activity of artemisinin was also greatly improved, suggesting that ART activity may be closely linked with the mitochondria. Given that mitophagy is closely linked to mitochondrial maintenance and status, it is therefore of interest to investigate potential links between ART treatment and mitophagy in cancer cells in order to investigate the possible role of ART in anticancer.

Hamacher-Brady et al. [23] first reported the effect of ART on autophagy induction via disrupting endolysosomal trafficking. It was found that ART induces perinuclear clustering of autophagosomes, early and late endosomes, and lysosomes. Lysosomes contain high levels of redox-active iron and iron-catalyzed lysosomal ROS production leads to mitochondrial outer membrane permeabilization and apoptosis in response to specific stimuli [23]. It is consistent with many studies [24], [25], [26] that ART treatment leads to mitochondrial ROS accumulation, disruption of mitochondrial membrane potential and induction of the intrinsic mitochondrial apoptosis. However, it remains unknown whether these damaged mitochondria could be degraded through mitophagy. Our previous studies showed [27] that ART causes autophagic degradation of ferritin and mitochondrial membrane permeabilization, but through lysosomal activation rather than disruption, which is accompanied with the promotion of vacuolar-type H^+^-ATPase (V-ATPase) assembly [27]. This give us a further indication that the damaged mitochondria could be degraded through mitophagy.

In this study, we demonstrated for the first time that ART induces mitophagy through the PINK1-dependent pathway. Notably, ART enhances the interaction of PINK1 and Parkin, which results in the recruitment of Parkin to the mitochondria and ultimately culminates in mitophagy. PINK1 is required for ART-induced mitophagy and mitophagy exerts its influence by stabilizing mitochondrial membrane potential, controlling oxidative stress and protecting the cell from apoptosis. Our study provides novel insight into the mitophagic effect of ART in cancer cells and suggests that mitophagy inhibition may be applied to enhance the anticancer activity of ART.

## 2 Materials and methods

### 2.1 Reagents and antibodies

Chloroquine diphosphate (CQ, Sigma, C6628). Tris [(1-benzyl-1H-1,2,3-triazol-4-yl) methyl] amine (TBTA), Tris (2-carboxyethyl) phosphine (TCEP), CuSO_4_ were purchased from Sigma. Rhodamine-azide was obtained from Jinglan Co. (Guangzhou, China) and biotin-azide was purchased from Thermo Fisher Scientific. The ART-probe (ART-P) was synthesized as previous description [20]. MitoSOX™ Red Mitochondrial Superoxide Indicator (M36008), JC-1 Mitochondrial Membrane Potential Dye (65-0851-38), MitoTracker™ Red (M7513), Pacific Blue™ Annexin V Apoptosis Detection Kit (A35122), ThiolTracker™ Violet (glutathione, T10095) were purchased from Thermo Fisher Scientific.

The following antibodies were used: autophagy-related gene 7 (ATG7) (2631), cytochrome c oxidase Ⅳ (COX Ⅳ) (4850), dynamin-related protein 1 (DRP1) (8570), phospho-DRP1 (Ser616) (3455), heat shock protein 60 (HSP60) (12165), lysosomal-associated membrane protein 1 (LAMP1) (9091), mitofusin 1 (MFN-1) (14739), MFN-2 (11925), Dynamin-like 120 kDa protein (OPA-1) (67589), PINK1 (6946), Parkin (4211), SQSTM1/P62 (5114), Ubiquitin (3936), were purchased from Cell Signaling Technology. α-tubulin (sc-23948), translocase of outer mitochondrial membrane 20 (TOMM20) (sc-11415), translocase of the inner membrane 23 (TIM23) (sc-514463) were purchased from Santa Cruz Biotechnology. LC3 (L7543) was purchased from Sigma.

### 2.2 Cell culture

HeLa cells were obtained from American Type Culture Collection (ATCC^®^ CCL-2™). All cell lines were maintained in DMEM (Sigma, D1152) containing 10% fetal bovine serum (HyClone, SV30160.03) in a 5% CO_2_ atmosphere at 37 ℃.

### 2.3 Detection of the intracellular localization of ART

The cells were treated with red fluorescent ART (20 μM, LynxTag-ART™AS_Red_, from BioLynx Technologies) in full DMEM for 30 min. Subsequently, cells were fixed with 4% formaldehyde for 15 min, permeabilized with 0.1% Triton X-100 for 10 min and blocked with 10% FBS. Cells were incubated with TOMM20 antibodies at 4 °C overnight, followed by incubation with the following Alexa Fluor^®^ 488 goat anti-rabbit (Thermo Fisher Scientific, A-11034) at 37 °C for 1 h. Cells were examined and recorded using a confocal microscope (Leica TCS SP8, Leica Microsystems, Germany) and representative cells were selected and photographed.

### 2.4 *In situ* labeling of ART-probe

Cells were cultured in six-well plates until 80–90% confluence was reached. As described before [20], ART-probe (20 μM) in 2 ml of medium with a final DMSO concentration of 1% was added, and the cells were incubated at 37 °C with 5% CO_2_ for 6 h. After treatment, the cells were lysed to obtain total cell lysates or harvested to isolate mitochondrial fraction according to the manufacture (Thermo Fisher Scientific, 89874). Equal amounts of the extracted proteins were then subjected to fluorescence labeling. The “click” reaction was done by adding Rhodamine B-azide (10 μM), TCEP (1 mM), TBTA (100 μM), and CuSO_4_ (1 mM) to the lysate, followed by 2 h incubation at room temperature. The labeled proteins were then acetone-precipitated and air-dried. The samples were then solubilized with 100 µL of 1× SDS loading buffer. Sample was separated with 4–20% gradient SDS-PAGE gel. Typhoon 9410 laser scanner (GE Healthcare) was used to obtain the gel images, which were analyzed by Image Quant software.

### 2.5 ART mitochondrial targets identification using chemical proteomics

Briefly, HeLa cells were cultured in 150 mm culture dish until 80% confluence was reached. After removal of culture medium and washing twice with PBS, ART-probe (20 μM) in 20 ml of medium with a final DMSO concentration of 1% was added to the cells, followed by incubation for 6 h. Control treatments were performed with culture medium containing 1% DMSO. The media were discarded after treatment, and then the cells were subjected to PBS wash and mitochondrial fraction was isolated according to the manufacturer's instructions (Thermo Fisher Scientific, 89874). Equal amounts of the extracted mitochondrial proteins were conjugated with the biotin tags separately via click chemistry, by adding biotin-azide (10 μM), TCEP (1 mM), TBTA (100 μM) and CuSO_4_ (1 mM) followed by 4 h shaking. The reacted proteins were then acetone-precipitated and air-dried. The pellet was re-solubilized with 1 ml of PBS containing 0.1% SDS and then added to 40 µL of Streptavidin beads, followed by 2 h incubation at room temperature with gentle mixing. Then, the pull-down samples were trypsin digested and identified by LC-MS/MS [28]. Subsequent gene ontology (GO) analysis for cellular component enrichment was conducted using Cytoscape 3.6.1 with ClueGO plugin.

### 2.6 Confocal microscopy

Cells were seeded on coverslips and cultured in 12-well plates overnight. The cells were subsequently treated at the indicated time points. After treatment, cells were fixed with 4% formaldehyde for 15 min, permeabilized with 0.1% Triton X-100 for 10 min and blocked with 10% FBS. Cells were incubated with various primary antibodies at 4 °C overnight, followed by incubation with the following secondary antibodies at 37 °C for 1 h, as appropriate: Alexa Fluor 405^®^ goat anti-mouse (Thermo Fisher Scientific, A-31553), Alexa Fluor 594^®^ goat anti-rabbit (Thermo Fisher Scientific, R37117), Alexa Fluor^®^ 594 goat anti-mouse (Thermo Fisher Scientific, A-11032). Cells were examined and recorded using a confocal microscope (Leica TCS SP8, Leica Microsystems, Germany) and representative cells were selected and photographed.

### 2.7 Measurement of mitochondrial superoxide

MitoSOX™ Red mitochondrial superoxide indicator is a novel fluorogenic dye for highly selective detection of superoxide in the mitochondria of live cells. It is live-cell permeant and is rapidly and selectively targeted to mitochondria. Once in mitochondria, MitoSOX™ Red reagent is oxidized by superoxide and exhibits red fluorescence. Cells were treated as indicated and then incubated with 5 μM MitoSOX™ reagent for 10 min at 37 °C, protected from light. The stained cells fluorescence emission was measured at 580 nm using flow cytometry.

### 2.8 Determination of mitochondrial membrane potential

JC-1 is a membrane permeable dye to determine mitochondrial membrane potential. It can selectively enter the mitochondrial where it reversibly changes color as membrane potentials increase. Upon membrane polarization, the formation of JC-1 aggregates cause shifts in emitted light from 530 nm to 590 nm when excited at 488 nm. The treated cells were labeled with 10 μg/ml JC-1 and fluorescence was determined using flow cytometry or immunofluorescence microscopy.

### 2.9 Measurement of cellular lactate levels

L (+)-Lactate is a metabolic compound in cells by the action of the enzyme lactate dehydrogenase (LDH). Lactate concentration is determined by an enzymatic assay, which results in a colorimetric (570 nm)/fluorometric (λex = 535 nm/λem = 587 nm) product, proportional to the lactate present. The treated cells were harvested and homogenized in 4 volumes of the lactate assay buffer. The samples were centrifuged at 13,000×*g* for 10 min to remove insoluble material. The master reaction mix of lactate enzyme, probe and assay buffer was added into samples. The samples were incubated for 30 min at room temperature, protected from light. The absorbance was measured using absorbance reader.

### 2.10 Small interfering RNA (siRNA) and transient transfection

The scrambled RNAi oligonucleotides and siRNAs targeting PINK1 (sc-44598, Santa Cruz Biotechnology) were transfected into HeLa cells using the DharmaFECT 4 Transfection Reagent (Dharmacon, T-2001-02) according to the manufacturer's protocol. After the designated treatment, cell lysates were prepared for western blotting.

### 2.11 Western blotting

At the end of the designated treatments, cells were lysed in Laemmli SDS buffer (62.5 mM Tris at pH 6.8, 25% glycerol, 2% SDS, phosphatase inhibitor (Pierce, 78428) and proteinase inhibitor cocktail (Roche Applied Science, 11836153001). An equal amount of protein was resolved by SDS-PAGE and transferred onto PVDF membrane. After blocking with 5% non-fat milk, the membrane was probed with designated primary and secondary antibodies, developed with the enhanced chemiluminescence method, and visualized with the ChemiDoc MP (BIO-RAD).

### 2.12 Immunoprecipitation

For immunoprecipitation, equal quantities of proteins were incubated with PINK1 antibody at 4 °C overnight followed by incubation with protein A/G agarose beads (Santa Cruz Biotechnology, sc-2003) for 3 h, after which immune complexes were collected by centrifugation. After washing 5 times in phosphate-buffered saline (PBS), samples were subjected to western blotting analysis.

### 2.13 Detection of viable and dead cells

Several methods were used to detect cell death quantitatively and qualitatively, which are (i) morphological changes under phase-contrast microscopy; (ii) Pacific Blue conjugate Annexin V staining for cell apoptosis. For Annexin V staining, the medium in each well was collected and cells were harvested with trypsin after treatment. Then, cell pellets obtained were resuspended in 1x annexin-binding buffer (A35136, Thermo Fisher Scientific) and labeled with 5 µL Pacific Blue™ Annexin V and 1 µL SYTOX^®^ AADvanced™ solution (5 μM) at room temperature for 30 min. Cell fluorescence was measured flow cytometry using 405 nm and 488 nm excitation. Ten thousand cells from each sample were analyzed with FACSCalibur flow cytometry (BD Bioscience, San Jose, CA) using CellQuest software.

### 2.14 Statistical analysis

All western blotting and image data presented are representatives from at least 3 independent experiments. The numeric data are presented as means ± SD from 3 independent experiments and analyzed using the Student's *t*-test.

## 3 Results

### 3.1 ART accumulates in mitochondria

**Fig. 1:**
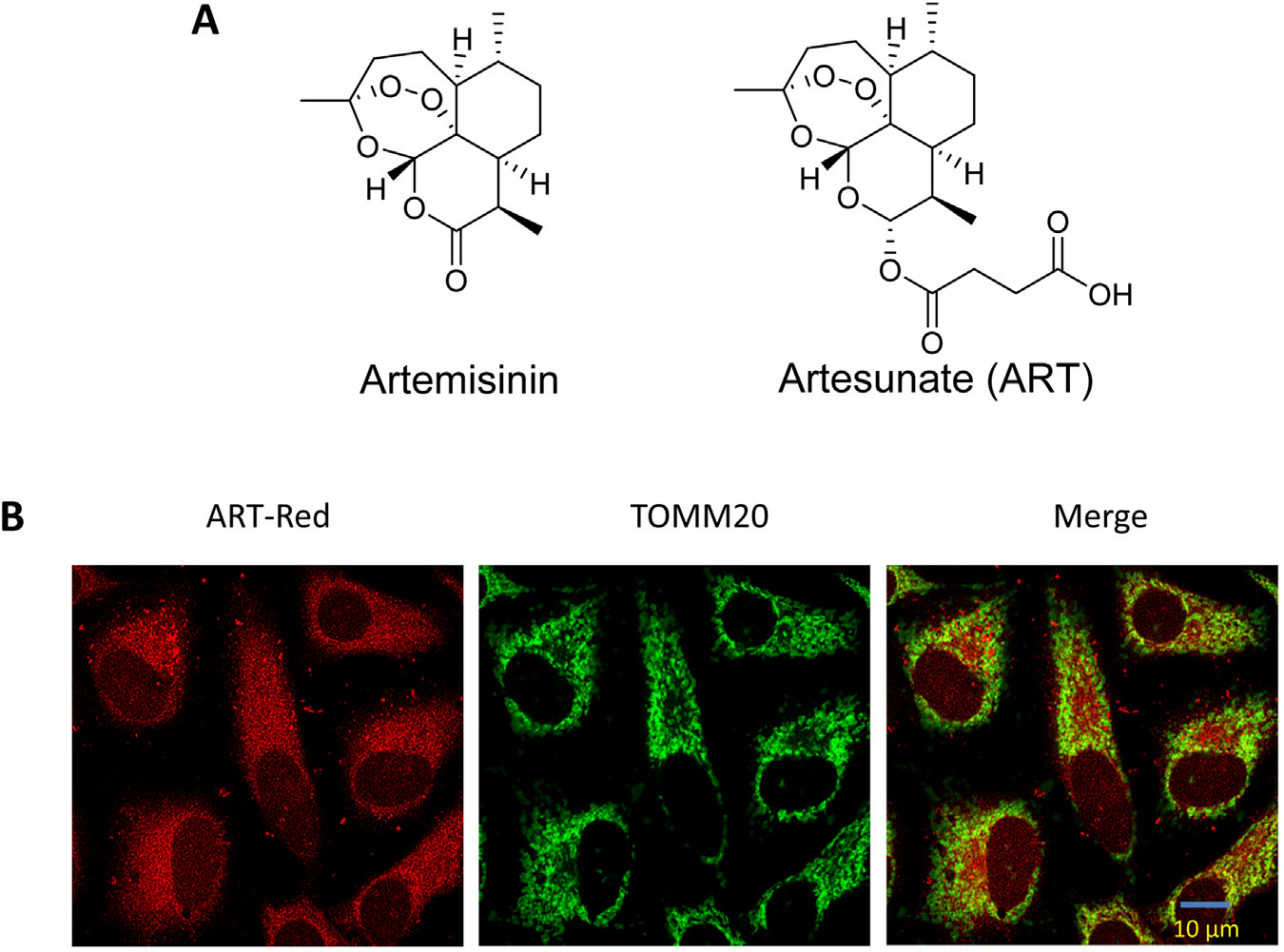
**Artesunate accumulates in the mitochondria. (A)** Chemical structures of artemisinin and artesunate (ART). **(B)** Fluorescence microscopy for the localization of ART in HeLa cells. The cells were treated with red fluorescent ART (20 µM) for 30 min and then immunostained with anti-translocase Of Outer Mitochondrial Membrane 20 (TOMM20) and green fluorescence dye. All images were analyzed using confocal microscopy. Scale bar: 10 µm.

Artemisinin, also known as qinghao su and its semi-synthetic derivatives are a group of drugs used against *Plasmodium falciparum*-induced malaria [29]. Chemically, artemisinin is a sesquiterpene lactone containing an unusual peroxide bridge, which is believed to be responsible for the drug's mechanism of action (Fig. 1**A**). ART, is a semi-synthetic derivative of artemisinin (Fig. 1**A**), has improved water solubility over the original Artemisinin [14]. ART is made from dihydroartemisinin (DHA) by reacting it with succinic acid anhydride in a basic medium.

Our previous studies showed that ART is activated by heme derived from hemoglobin digestion by *Plasmodium falciparum* and then exerts its anti-malarial activity [20], [21]. When ART is targeted to mitochondria (the primary site of heme synthesis) using a mitochondria-directing derivative-ART-TPP (triphenylphosphoniumbromide), ART activity is shown to be highly improved [22]. Here, we utilized LynxTag-ART™ AS_Red_, a red fluorescence-tagged ART, to investigate its cellular localization. As shown in Fig. 1**B**, ART was found to be predominantly localized in mitochondria, as evidenced by the colocalization of ART (red) with the translocase of the outer mitochondrial membrane 20 (TOMM20, green).

### 3.2 ART induces cell apoptosis through mitochondrial pathway

**Fig. 2:**
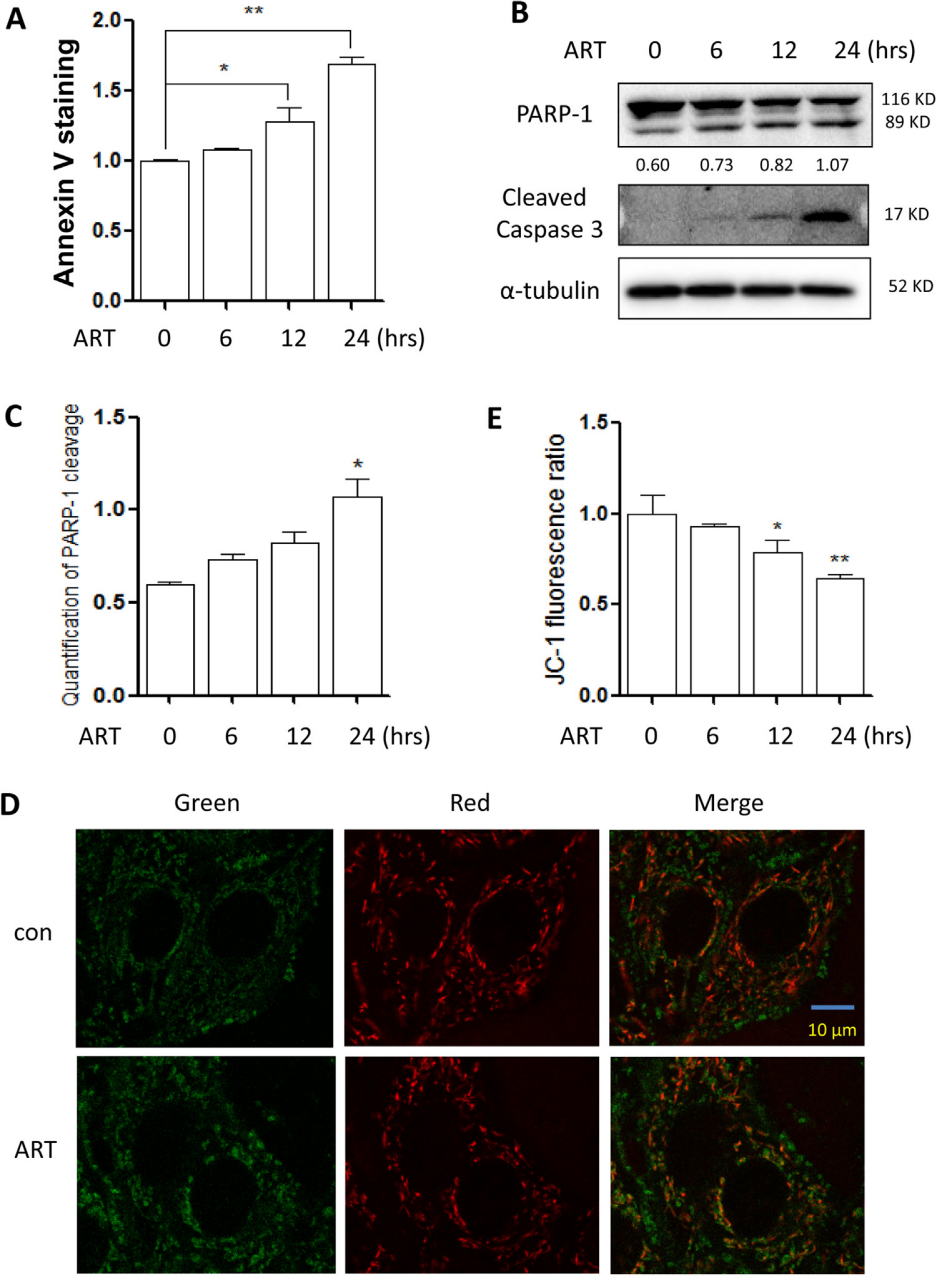
**ART induces apoptosis via the mitochondrial pathway in HeLa cells. (A)** Cell viability of ART-treated HeLa cells. The cells were treated with 20 µM ART from 0 to 24 h. Cells were stained using Annexin V-Pacific Blue and cell fluorescence was measured by flow cytometry. Data are presented as mean ± SD (* *P* < 0.05, ** *P* < 0.01). **(B)** Detection of ART-induced apoptosis in HeLa cells. Cell lysates obtained from treatments indicated in (A) were prepared and subjected to western blotting analysis using antibodies against PARP-1 and cleaved-Caspase 3. α-tubulin was used as the loading control. **(C)** The level of PARP-1 cleavage was also quantified and statistically analyzed. * *P* < 0.05. **(D)** Fluorescence detection of the mitochondrial membrane potential in ART-treated HeLa cells. The cells were first treated with ART (10 µM, 12 h) and then stained with the dye for mitochondrial membrane potential, JC-1. Fluorescence of J-aggregates (red) and J-monomers (green) was detected under confocal microscope. Scale bar: 10 µm. **(E)** Quantification of the mitochondrial membrane potential in ART-treated HeLa cells. The ratio of J-aggregates to J-monomers as captured from (C) was calculated as indicated and statistically analyzed. * *P* < 0.05, ** *P* < 0.01.

**Fig. S1:**
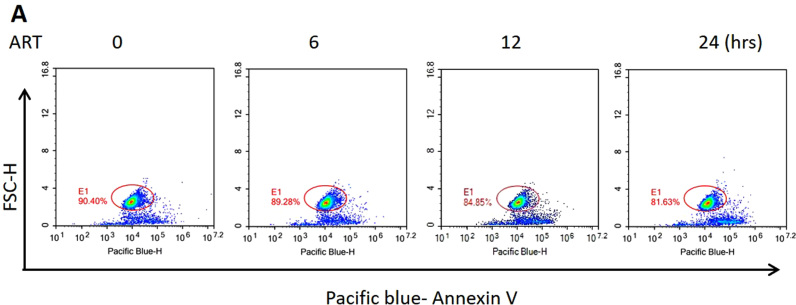
**Suppl Fig.** 1**. Flow cytometric analysis of HeLa cells using apoptosis detection kit.** HeLa cells were treated with ART (20 μM) for different time and then harvested for staining with Annexin V-Pacific Blue. Cell fluorescence was measured using flow cytometry.

We first evaluated the effect of ART on cell apoptosis in HeLa cells. Annexin V staining results showed that ART treatment significantly increase apoptotic cell death in a time-dependent manner (Fig. 2**A** and Suppl Fig. 1A). It was also confirmed by the increased cleavage of Poly [ADP-ribose] polymerase 1 (PARP-1) and Caspase 3 (Figs. 2**B** and 2**C**). We then determined the mitochondrial membrane potential in ART-treated cells. As shown in Fig. 2**D**, potential-dependent staining of the mitochondria was performed using JC-1. Regions of high mitochondrial polarization are indicated by red fluorescence due to J-aggregate formation by the concentrated dye. Depolarized regions are indicated by the green fluorescence of the JC-1 monomers. It was observed that ART decreased mitochondrial membrane potential. Accordingly, the ratio of J-aggregates to J-monomers was also calculated and it was significantly decreased with time (Fig. 2**E**).

### 3.3 ART mainly targets mitochondrial proteins

**Fig. 3:**
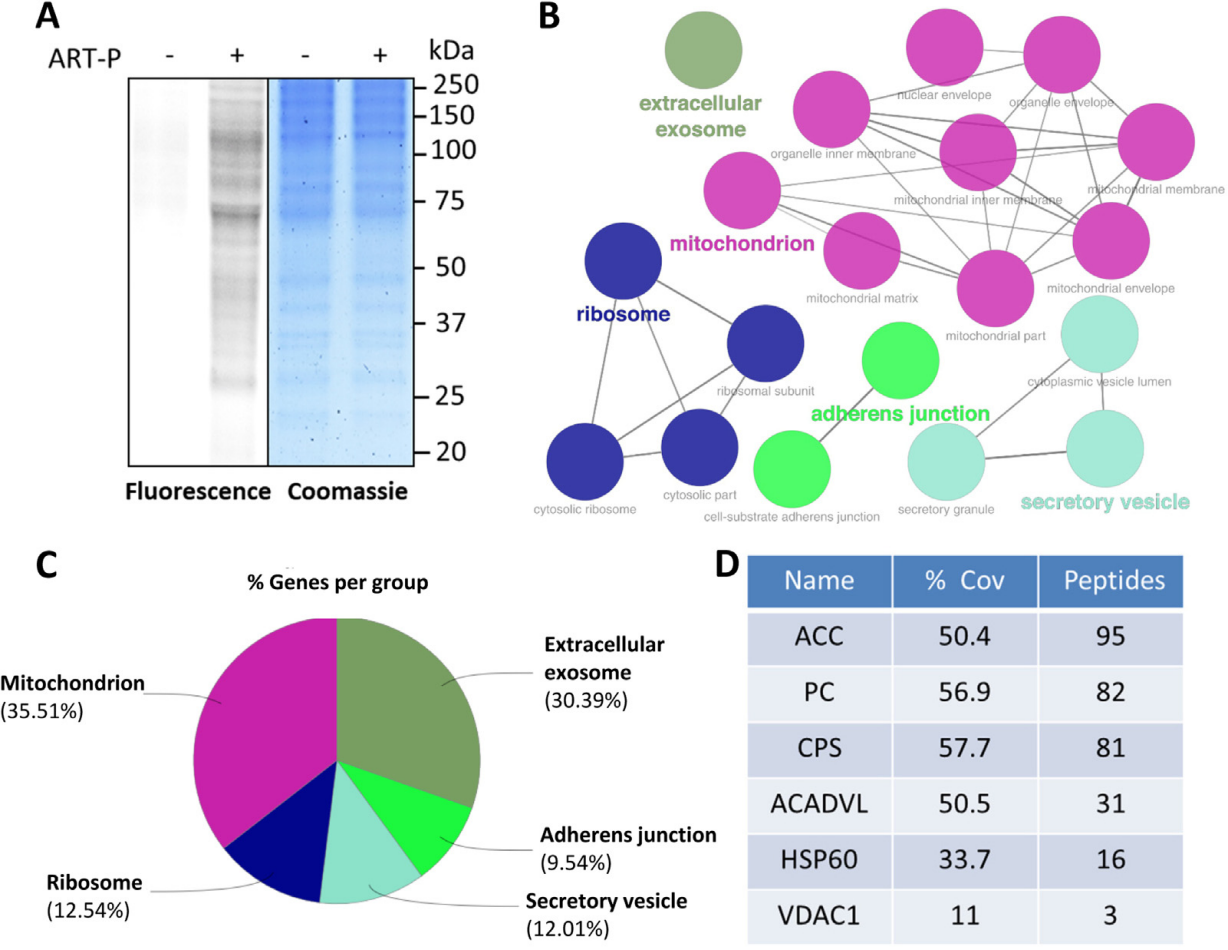
**ART targets mitochondrial proteins. (A)** Fluorescence labeling of the mitochondrial fraction of HeLa cells labeled with ART-probe. The cells were treated with ART-probe (20 µM) for 6 h and then the mitochondrial fraction was enriched and visualized by “click” conjugation to the Rhodamine B-azide tag followed by SDS-gel separation and fluorescence scanning. **(B)** Network of the cellular component enrichment from gene ontology (GO) analysis. The identified direct protein targets of ART-probe show enrichment in mitochondria and mitochondria associated cellular components. **(C)** Pie chart of GO analysis for cellular component enrichment using the list of identified direct protein targets of ART-probe. As highlighted in (B), majority of the proteins are from the mitochondria. **(D)** The representative mitochondrial proteins identified by ART-probe in HeLa cells. ART-P, ART-probe; ACC, acetyl-CoA carboxylase 1; PC, pyruvate carboxylase; CPS, carbamoyl-phosphate synthase; ACADVL, very long-chain specific acyl-CoA dehydrogenase; HSP60, heat shock protein 60 kDa; VDAC1, voltage-dependent anion-selective channel protein 1; % Cov, percent protein sequence coverage with the identified peptides.

To profile and identify the mitochondrial targets of ART, we utilized ART-probe, a chemically engineered artemisinin with a clickable alkyne tag attached [20]. We first labeled HeLa cells with ART-probe and then prepared the mitochondrial fraction. The alkyne tagged mitochondrial proteins can be further appended with a fluorescent dye Cy3-azide through “click” chemistry. Only the covalent binding targets of ART-probe can be visualized through SDS-polyacrylamide gel electrophoresis (PAGE) and followed by fluorescence scanning (Fig. 3**A**).

Next, we went on to identify the covalent binding targets of ART using mass spectrometry. HeLa cells were first labeled with ART-probe and then subjected to mitochondrial extract preparation, followed by biotin labeling of the alkyne tag. The ART targets were affinity purified by streptavidin beads and identified with tandem mass spectrometry. A total of 442 proteins were identified as direct targets of ART and documented in Suppl Table 1. A subsequent GO analysis for cellular component enrichment reviewed that majority of the direct protein targets originates from the mitochondria (Fig. 3B and C). Among the identified mitochondrial proteins from our target list, we highlighted 6 representative mitochondrial proteins as shown in Fig. 3D, which are mitochondrial membrane proteins or enzymes for energy metabolism [30]. These results further support our notion that ART acts mainly in the mitochondria, and could exert mitochondria-specific processes that lead to our observed ART-induced cell apoptosis.

### 3.4 ART induces mitochondrial fission and mitophagy in HeLa cells

**Fig. S2:**
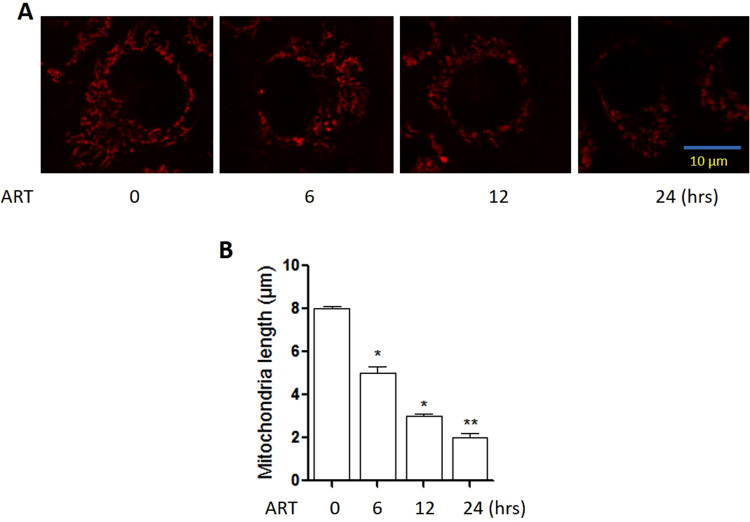
**Suppl Fig.** 2**. ART induces mitochondrial fission. (A)** Fluorescence microscopy of the mitochondrial length in ART-treated HeLa cells. The cells were exposed to 10 μM of ART for different durations as indicated. Mitochondria were stained using MitoTracker and observed under confocal microscope. Scale bar: 10 µm. **(B)** Average mitochondrial length in ART-treated HeLa cells. Three independent experiments are performed as shown in (A). Data are presented as mean ± SD (* *P* < 0.05 ** *P* < 0.01)..

Increasing evidence indicates that mitochondrial fission promotes the initiation of mitochondrial apoptosis [31]. We examined the effect of ART on mitochondrial fission by staining HeLa cells with the mitochondrion-selective probe call Mitotracker. ART treatment significantly increased mitochondrial fission and markedly decreased the average mitochondria length in a time-dependent manner (Suppl Fig. 2). Consistently, in ART-treated cells, the level of phosphorylated DRP1 was enhanced (Suppl Figs. 5A and 5B), indicating the promotion of mitochondrial fission.

**Fig. 4:**
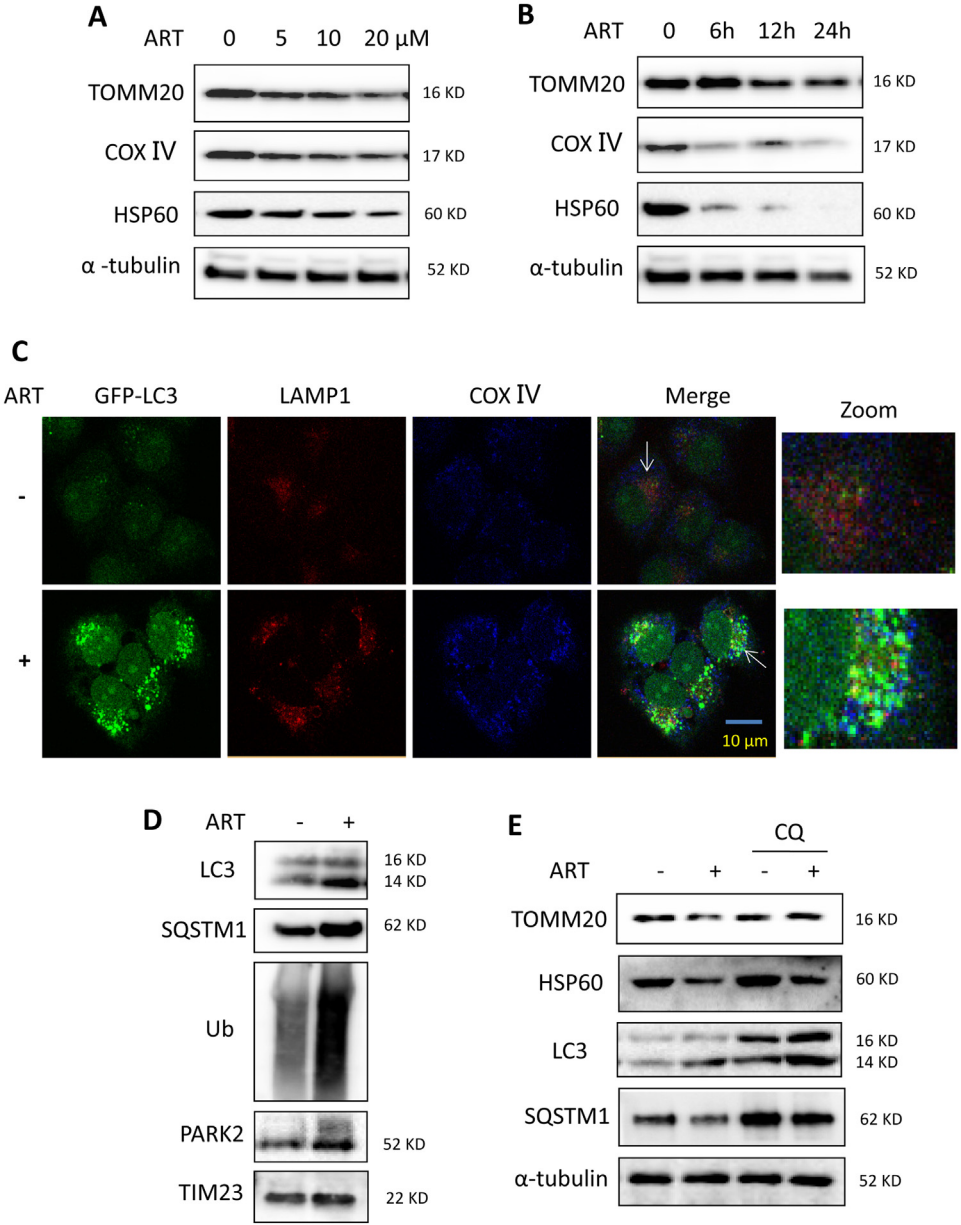
**ART induces mitophagy in HeLa cells. (A)** Western blot analysis of mitochondrial protein change in ART-treated HeLa cells at different concentration of ART. Cells were treated with different dosages of ART (5, 10 or 20 μM) for 12 h. Cell lysates were then prepared and subjected to western blotting analysis for mitochondrial proteins TOMM20, COX Ⅳ and HSP60. α-tubulin was used as the loading control. **(B)** Western blot analysis of mitochondrial protein change in ART-treated HeLa cells at different duration. The cells were treated with ART (10 μM) for different time (6, 12 or 24 h) and analysis as per (A). **(C)** Fluorescence imaging of ART-treated HeLa cells to identify mitophagy induction. HeLa cells stably expressing GFP-LC3 were treated with ART (10 μM, 12 h). After immunostaining with LAMP1 (Alexa Fluor 594, red) and COX Ⅳ (Alex Fluo 405, blue), cells were examined under confocal microscopy. Scale bar: 10 µm. **(D)** Western blot analysis for Parkin and mitophagy levels in ART-treated HeLa cells. The cells stably expressing Parkin were treated with ART as indicated, and PARKIN, LC3, SQSTM1 and ubiquitin (Ub) levels in the mitochondrial fraction were determined by western blotting. TIM23 was used as a representative of mitochondrial fraction. **(E)** Mitophagy activity in ART and chloroquine (CQ)-treated HeLa cells. The cells were treated with ART (10 μM, 12 h) in the presence or absence of autophagy inhibitor chloroquine (CQ, 25 μM). Cell lysates were prepared for western blotting to detect for TOMM20, HSP60, LC3 and SQSTM1. α-tubulin was used as the loading control.

Our previous studies [27] showed that ART promotes lysosomal function and ferritin degradation, leading to mitochondrial reactive oxygen species (mROS) production and eventual cell apoptosis. As mitophagy is mediated by lysosomes, the elevated lysosomal function suggests that mitochondria damaged by ART could be eliminated through mitophagy [31]. We thus examined whether ART affects mitophagy level changes in HeLa cell. As shown in Figs. 4**A** and 4**B**, we treated HeLa cells with different dosages of ART at different time points. Western blotting analysis showed that ART treatment decreased mitochondrial TOMM20, cytochrome c oxidase Ⅳ (COX Ⅳ) and HSP60 protein levels in a time- and dose-dependent manner, suggesting that ART treatment leads to mitochondrial damage.

Furthermore, we performed confocal imaging to monitor the formation of autophagosomes and found that in HeLa cells stably expressing GFP-LC3, ART treatment increased the colocalization of GFP-LC3, LAMP1 and COX Ⅳ (Fig. 4**C**), indicating the enhanced fusion of autophagosomes, lysosomes and mitochondria. In addition, we prepared the mitochondrial fraction of ART-treated HeLa cells with Parkin overexpression and found that ART treatment enhanced the levels of Parkin in mitochondria and the ubiquitination levels of mitochondrial proteins (Fig. 4**D**). Meanwhile, ART also increased the levels of LC3-Ⅱ (autophagosome marker) and SQSTM1/P62 (a selective autophagy adaptor) in mitochondria (Fig. 4**D**). This indicates that ART treatment promotes the translocation of Parkin to mitochondria, which recruits autophagy receptors SQSTM1 and LC3 for autophagosome formation [12].

**Fig. S3:**
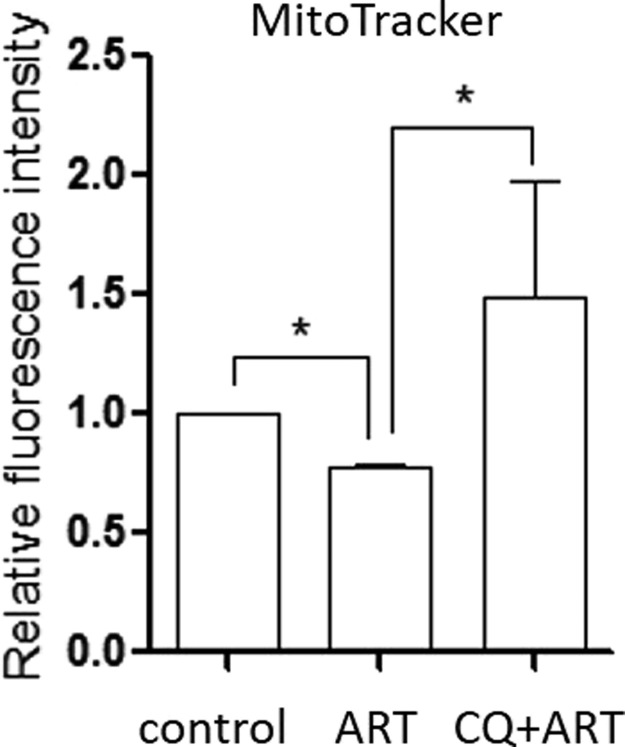
**Suppl Fig.** 3**. ART induces mitophagy in HeLa cells.** HeLa cells were treated with ART (10 μM) for 12 h in the presence or absence of autophagy inhibitor chloroquine (CQ, 25 μM). The cells were then stained using MitoTracker Red for mitochondria detection. Flow cytometry was performed to analyze cell fluorescence intensity. * *P* < 0.05..

To explore whether those damaged mitochondria by ART were eliminated by mitophagy, we treated cells with chloroquine (CQ), an inhibitor of the lysosomal pH gradient which prevents lysosome-mediated autophagic degradation. As shown in Fig. 4**E**, western blotting showed that ART modestly increased LC3-Ⅱ accumulation, which was further increased by CQ treatment. On the contrary, the level of SQSTM1 was decreased which demonstrates an expected increase in autophagic flux. The ART-induced decrease in TOMM20 and HSP60 levels was largely reversed by CQ treatment (Fig. 4**E**), suggesting that ART treatment increases autophagic flux level in HeLa cells. Additionally, we performed a flow cytometry-based approach to determine mitophagic flux using MitoTracker, a widely used mitochondria-selective probe, in combination with autophagy inhibitors [32]. As shown in Suppl Fig. 3, ART treatment resulted in a decrease of cell fluorescence, which could be reversed by CQ, indicating an enhanced mitophagy flux. The above findings demonstrate that ART induces mitophagy in HeLa cells.

### 3.5 The involvement of the PINK1-dependent pathway in ART-induced mitophagy

**Fig. 5:**
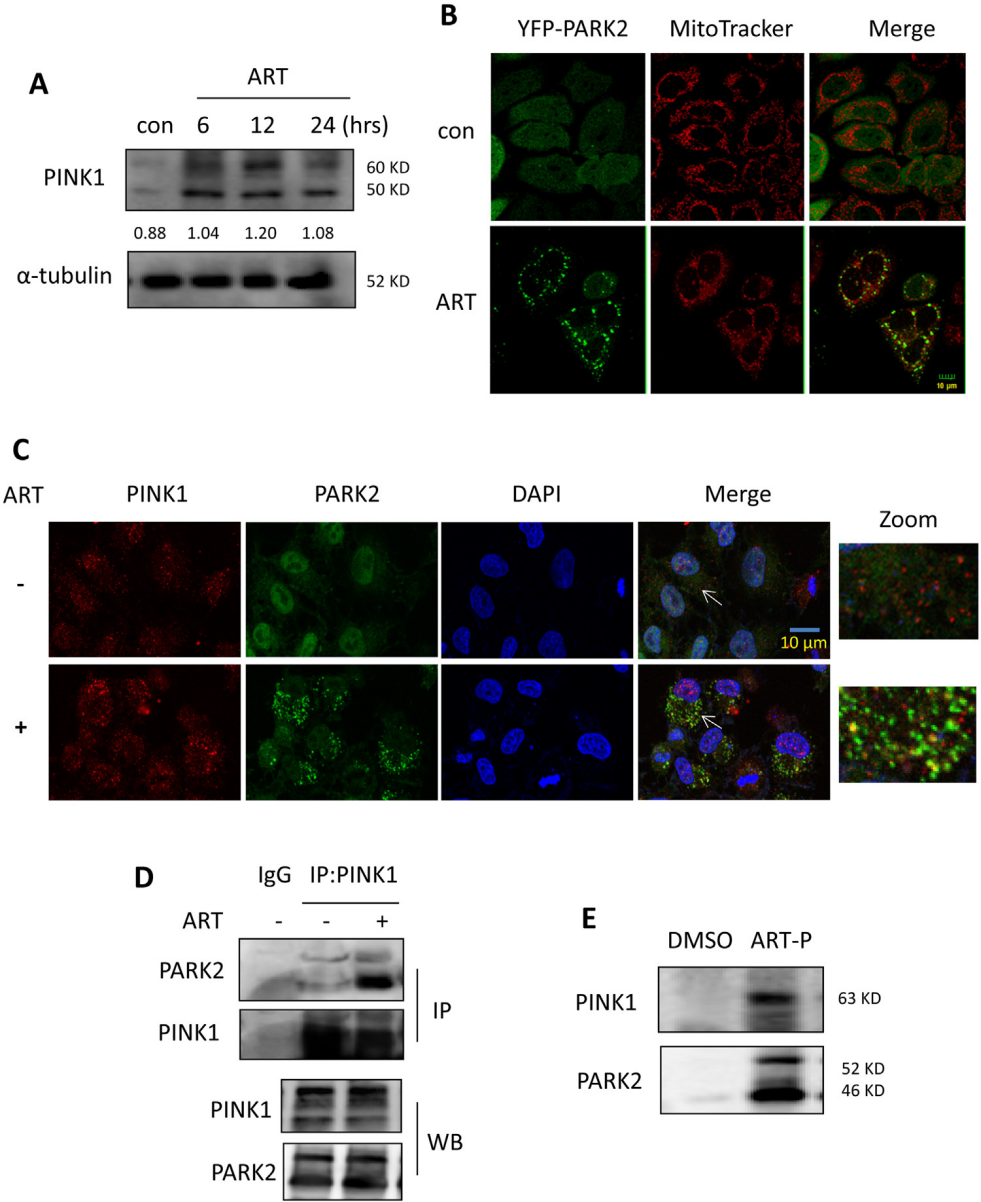
**ART triggers the PINK1-dependent mitophagy. (A)** Western blot analysis for PINK1 level in ART-treated HeLa cells. The cells stably expressing Parkin were treated with ART (10 μM) for different periods of time as indicated. Cell lysates were prepared and analyzed for PINK1 by western blotting using the anti-PINK1 antibody. **(B)** Fluorescence microscopy to identify the recruitment of Parkin to the mitochondria in HeLa cells. The cells stably expressing YFP-Parkin were treated with ART (10 μM, 12 h) and then stained with MitoTracker Red to detect the mitochondrial. Confocal microscopy was performed for evaluation. Scale bar: 10 µm. **(C)** Western blot analysis of PINK1 and Parkin colocalization in HeLa cells. After immunostaining with PINK1 (Alexa Fluor 594, red) and DAPI (blue), YFP-Parkin expressing cells were examined by confocal microscopy. Scale bar: 10 µm. **(D)** Western blot analysis of Pink1 and Parkin interaction in ART-treated HeLa cells using immunoprecipitation. The cell lysates were prepared and subjected to immunoprecipitation using anti-PINK1 antibody. Associated Parkin was detected using immunoblotting. **(E)** Interaction of PINK1 and Parkin with ART in HeLa cells. The cells stably expressing Parkin were labeled with ART-probe (10 μM) for 12 h. Cell lysates were prepared and visualized by “click” conjugation to the biotin-azide tag followed by SDS-gel separation. Western blotting was performed to validate ART-targeted PINK1 and Parkin proteins.

The PINK1-dependent pathway plays an important role in controlling mitophagy [11]. PINK1 recruits Parkin to the mitochondrial surface and ubiquitinates numerous mitochondrial proteins, leading to the onset of mitophagy [11]. We therefore examined the effect of ART on PINK1 expression and found that in HeLa cells stably expressing Parkin, ART treatment increased the level of PINK1 (Fig. 5**A**) in a time-dependent manner. As a result, the recruitment of Parkin (green) to mitochondria (MitoTracker, red) was promoted in ART-treated cells (Fig. 5**B**), leading to the ubiquitination of mitochondrial proteins.

Next, we performed immunofluorescence microscopy to investigate the interaction of PINK1 with Parkin by ART and found that PINK1 (red) markedly colocalized with Parkin (green) in the mitochondria in ART-treated cells (Fig. 5**C**), although a portion of PINK1 proteins localized to the nucleus. In addition, an immunoprecipitation assay was also performed and showed that ART treatment enhanced the interaction of PINK1 with Parkin (Fig. 5**D**). These findings indicate that ART treatment stabilizes the expression of PINK1 at the mitochondrial surface and in turn recruits Parkin to mitochondria.

To validate whether ART directly regulates the PINK1-dependent pathway, we used a synthesized cell permeable ART-probe (ART-P) with an alkyne moiety [20], which can be tagged with biotin-azide for affinity enrichment of the direct-binding protein targets of ART in situ. HeLa cells stably expressing Parkin were first labeled with the ART-probe and then cell lysates were prepared and reacted with biotin-azide through “click” chemistry followed by SDS-PAGE. As shown in Fig. 5**E**, ART-probe directly bound to PINK1 and Parkin in the cells, suggesting that PINK1 and Parkin are both molecular targets of ART.

### 3.6 Knockdown of PINK1 impairs ART-induced mitophagy

**Fig. 6:**
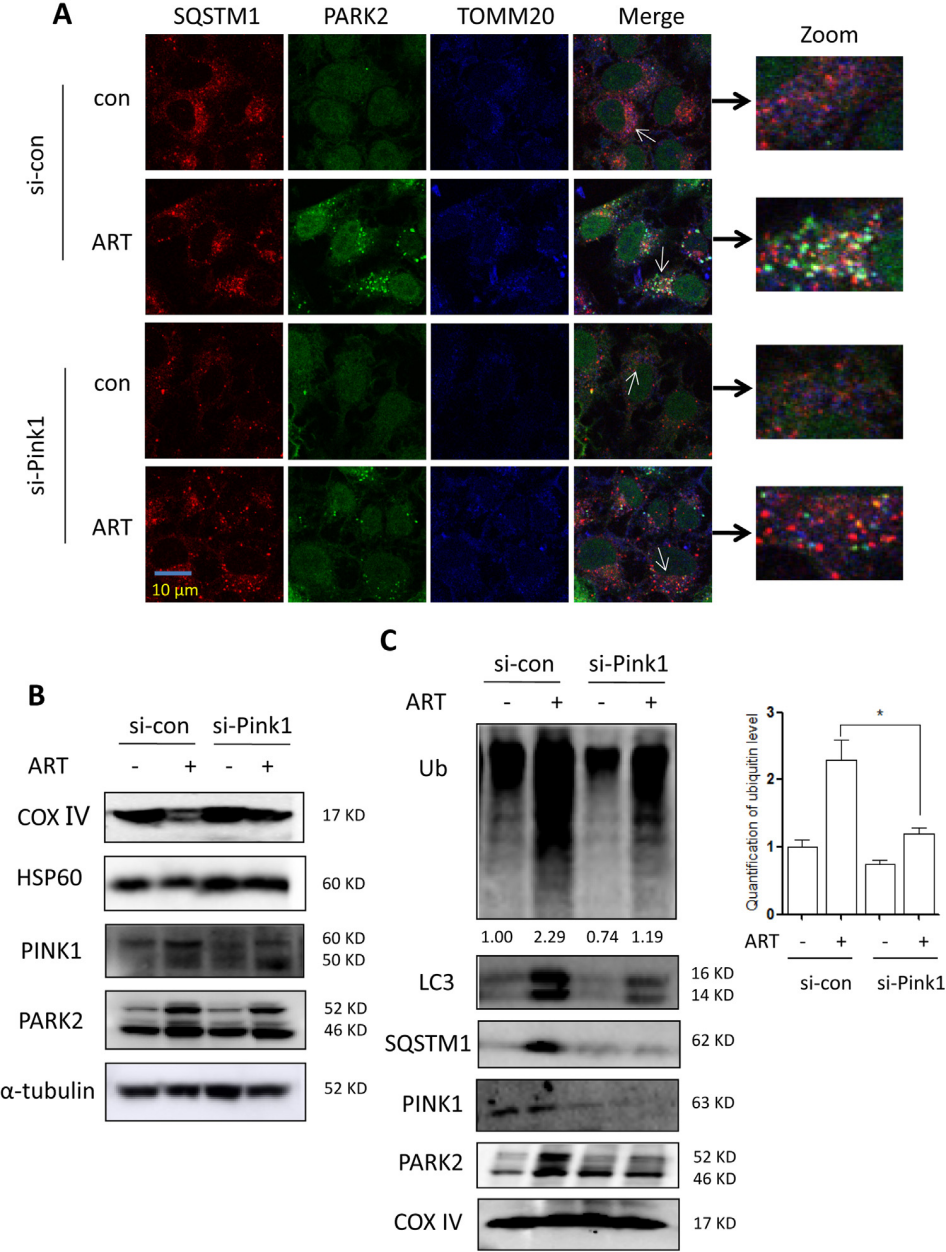
**Knockdown of PINK1 blocks ART-induced mitophagy. (A)** Fluorescence microscopy of Parkin recruitment to the mitochondria in ART-treated HeLa cells after PINK1 knockdown. The cells stably expressing YFP-Parkin were first transiently transfected with a nonspecific siRNA or the PINK1-specific siRNA and then cells were subsequently treated with 10 μM ART for 12 h. After immunostaining with SQSTM1 (Alexa Fluor 594, red) and TOMM20 (Alexa Fluor 405, blue), cells were examined using confocal microscopy. Scale bar: 10 µm. **(B)** Western blot analysis of mitochondrial proteins in ART-treated HeLa cells after PINK1 knockdown. After treatment, cell lysates were prepared and immunoblotted for COX Ⅳ, HSP60, PINK1 and Parkin levels. α-tubulin was used as the loading control. **(C)** Western blot analysis of mitophagy proteins in the mitochondrial fractions of ART-treated HeLa cells after PINK1 knockdown. The cells were treated as indicated and mitochondrial fractions were then prepared and subjected to western blotting analysis. The level of ubiquitinated proteins was quantified and statistically analyzed. COX Ⅳ was used as a representative of mitochondrial fraction. * *P* < 0.05.

**Fig. S4:**
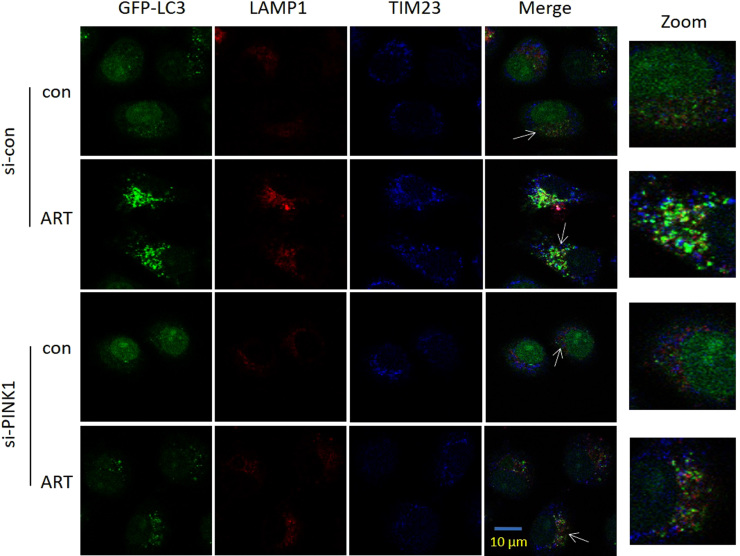
**Suppl Fig.** 4**. Knockdown of PINK1 impairs ART-induced mitophagy.** HeLa cells stably expressing GFP-LC3 were first transiently transfected with a nonspecific siRNA or the PINK1-specific siRNA. The cells were subsequently treated with 10 μM ART for 12 h. After immunostaining with LAMP1 (Alexa Fluor 594, red) and TIM23 (Alexa Fluor 405, blue), cells were examined using confocal microscopy. Scale bar: 10 µm.

To further confirm the role of the PINK1-dependent pathway in mitophagy induction by ART, we used siRNA to knock down PINK1 expression. In Fig. 6**A**, confocal imaging showed that knockdown of PINK1 markedly decreased ART-induced colocalization of Parkin, SQSTM1 and TOMM20, indicating reduced recruitment of Parkin to mitochondria. An immunofluorescence assay (Suppl Fig. 4) revealed that knockdown of PINK1 significantly reduced ART-induced colocalization of GFP-LC3, LAMP1 and translocase of the inner membrane 23 (TIM23), suggesting the decreased fusion of autophagosomes, lysosomes and mitochondria. Consistently, western blotting results (Fig. 6**B**) showed that when PINK1was knocked down, the reduction of mitochondrial COX Ⅳ and HSP60 protein levels following ART treatment was blocked. In the mitochondrial fraction, knockdown of PINK1 impaired the ART-induced increase in Parkin, SQSTM1, LC3 and ubiquitin levels in the mitochondria of the HeLa cells (Fig. 6**C**). This data indicates that ART treatment fails to induce mitophagy when PINK1 was knocked down, and that ART-induced mitophagy is dependent on the PINK1 pathway.

**Fig. S5:**
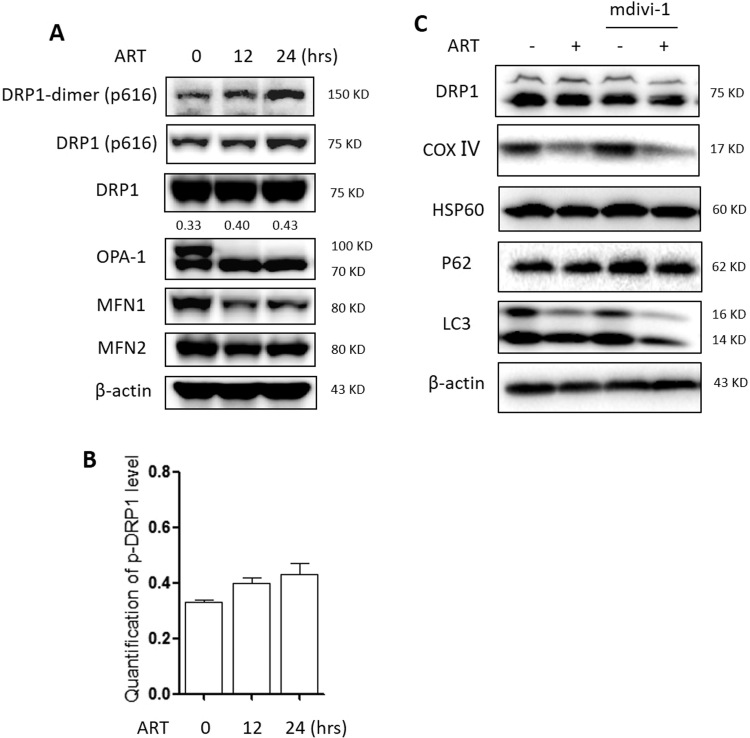
**Suppl Fig.** 5**. ART induces mitochondrial fission. (A)** Western blot analysis of mitochondrial fission markers in ART-treated HeLa cells. The cells were treated with ART (10 μM) for 12 or 24 h as indicated. The levels of DRP1, OPA-1 and MFN in cells were determined by immunoblotting. **(B)** The level of phosphorylated DRP-1 was quantified. **(C)** Mitophagy level in ART-treated HeLa cells after DRP1 inhibition by mdivi-1. HeLa cells were treated with ART in the presence or absence of mdivi-1 (5 μM) for 12 h. Cell lysates were prepared and subjected to western blotting analysis.

**Fig. S6:**
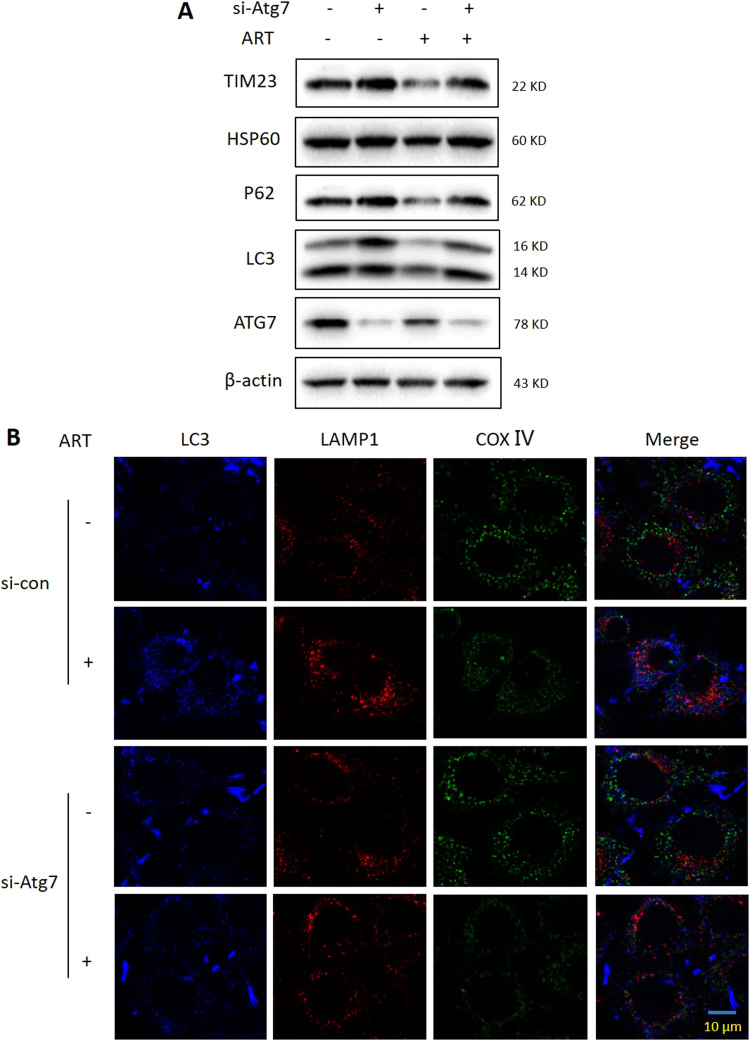
**Suppl Fig.** 6**. ATG7 depletion suppresses mitophagy. (A)** Western blot analysis of mitophagy levels in ART-treated HeLa cells after Atg7 knockdown. The cells were first transiently transfected with a nonspecific siRNA or the Atg7-specific siRNA and were subsequently treated with 10 μM ART for 12 h. Cell lysates were then prepared and subjected to western blotting analysis for mitochondrial proteins TIM23, HSP60, LC3, P62 and ATG7. β-actin was used as the loading control. **(B)** Fluorescence microscopy of mitophagy activity in ART-treated HeLa cells after Atg7 knockdown. The cells were prepared for immunostaining with LC3 (Pacific Blue), LAMP1 (Alexa Fluor 594, red) and COX Ⅳ (Alex Fluo 488, green) and then examined under confocal microscopy. Scale bar: 10 µm.

In addition, we investigated whether mitochondrial dynamics affects mitophagy level changes. As shown in Suppl Fig. 5B, in the presence of mdivi-1 (a pharmacological inhibitor of DRP1), there was no any alteration in mitophagy level by ART. Conversely, in Atg7-knockdown cells, ART failed to induce autophagy and the decrease of mitochondrial HSP60 and TIM23 by ART was also blocked (Suppl Fig. 6A). Confocal imaging showed that the enhanced fusion of autophagosomes, lysosomes and mitochondria by ART was also attenuated when Atg7 was knocked down. The above results suggested that ATG machinery is essential for the mitophagy induction by ART.

### 3.7 Mitophagy inhibition leads to changes of mitochondrial function

**Fig. 7:**
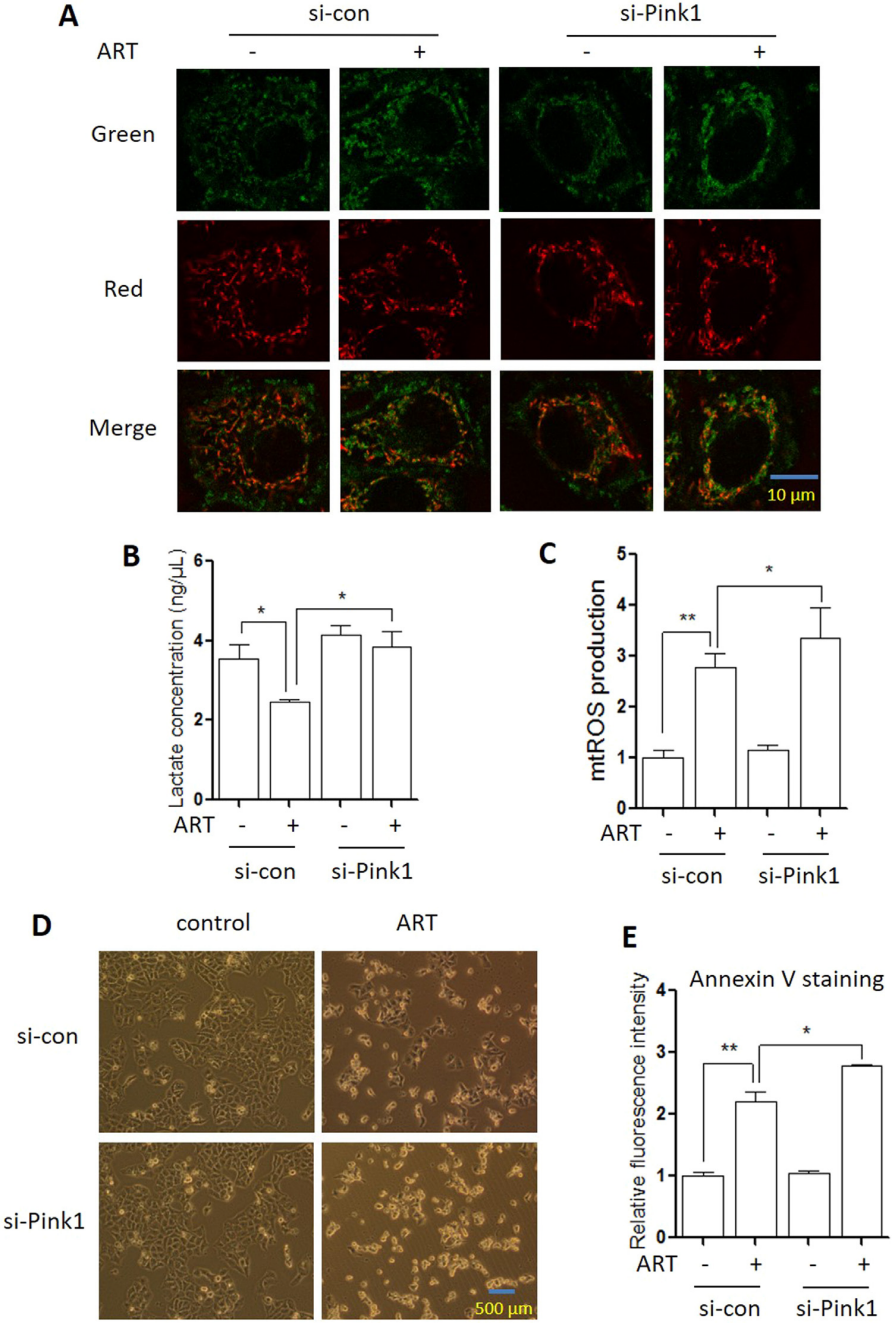
**Mitophagy suppression causes alterations in mitochondrial functions. (A)** Mitochondria membrane potential in ART-treated HeLa cells after PINK1 knockdown. The cells were transiently transfected with the PINK1-specific siRNA and then treated with ART (10 µM, 12 h). Cells were incubated with JC-1 (10 μg/ml) to assess mitochondrial membrane potential as described. Fluorescence of J-aggregates (red) and J-monomers (green) was detected under confocal microscope. Scale bar: 10 µm. **(B)** Glycolysis level of ART-treated HeLa cells after PINK1 knockdown. The cells were treated as indicated in (A). Cellular lactate levels were measured in cells as reported. The numeric data was analyzed using Student's *t*-test. * *P* < 0.05, ** *P* < 0.01. **(C)** Mitochondrial ROS level in ART-treated HeLa cells after PINK1 knockdown. The cells were treated as indicated in (A). Mitochondrial ROS were measured in the cells as reported. The numeric data was analyzed using Student's *t*-test. * *P* < 0.05, ** *P* < 0.01. **(D)** Effects of PINK1 knockdown on ART-induced cell death. HeLa cells were transiently transfected with a nonspecific siRNA or the PINK1-specific siRNA. After 72 h, cells were treated with ART (20 μM) for 24 h and morphological changes of HeLa cells with respective treatments were examined and photographed with an inverted microscope. Scale bar: 500 µm. **(E)** Cellular apoptosis of ART-treated HeLa cells after PINK1 knockdown. The cells reflected in (D) were harvested and resuspended in 1x annexin-binding buffer. Cells were then labeled with Pacific Blue™ Annexin V (5 µL) and cell fluorescence emission was measured. The numeric data was analyzed using Student's *t*-test. * *P* < 0.05, ** *P* < 0.01.

**Fig. S7:**
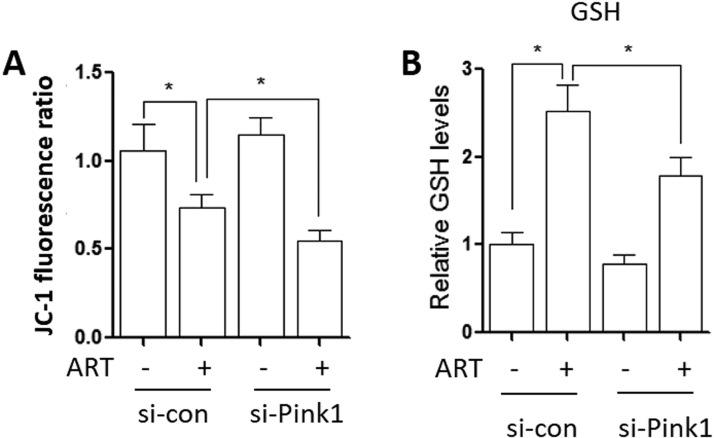
**Suppl Fig.** 7**. Inhibition of mitophagy alters mitochondrial function. (A)** Mitochondria membrane potential of ART-treated HeLa cells after PINK1 knockdown. The cells were first transiently transfected with the PINK1-specific siRNA and then probed with JC-1 (10 μg/ml) to assess mitochondrial membrane potential. Flow cytometry was performed to determine cell fluorescence. * *P* < 0.05 **(B)** Glutathione levels in ART-treated HeLa cells after PINK1 knockdown. The cells were first treated as per (A) and incubated with ThiolTracker™ Violet dye (20 µM) at 37 °C for 30 min. Cell fluorescence was measured by flow cytometry at 405 nm excitation. * *P* < 0.05.

To test whether ART-induced mitophagy influences mitochondrial dynamics, we first measured mitochondrial membrane potential changes using the JC-1 assay. JC-1 dye exhibits potential-dependent accumulation in mitochondria, indicated by a fluorescence emission shift from green (535 nm) to red (595 nm). As shown in (Fig. 7**A** and Suppl Fig. 7A), ART treatment significantly increased mitochondrial depolarization, which was indicated by a decrease in the red/green fluorescence intensity ratio. When PINK1 was knocked down, depolarized regions indicated by the green fluorescence of the JC-1 monomers were further increased. A significant decrease of red to green fluorescence was observed in ART-treated cells with PINK1 knockdown (Suppl Fig. 7A). This suggests that mitophagy by ART stabilizes the mitochondrial membrane potential.

Next, we determined the effect of mitophagy on energy metabolism. As widely known, mitochondria generates most of the cell's supply of adenosine triphosphate (ATP), used as a source of chemical energy [33]. As shown in Fig. 7**B**, ART treatment decreased the levels of cellular lactate and this decrease could be reversed when PINK1 was knocked down, indicating that mitophagy reduces the rate of glycolysis and enhances oxidative phosphorylation for energy.

Furthermore, cellular redox status was also determined in ART-treated cells. The major source of ROS is from cellular mitochondrial respiration, with 0.2% of oxygen consumed being normally converted into superoxide in a quiescent state [34]. Consequently, superoxide causes oxidative stress and may lead to the decline in mitochondrial functions. As shown in Fig. 7**C**, ART treatment increased the generation of mROS; when PINK1 was knocked down, more mitochondrial ROS was produced in ART-treated cells. On the contrary, the antioxidant glutathione (GSH) was significantly reduced by ART under PINK1 knockdown (Suppl Fig. 7B). This indicates that mitophagy maintains the cells’ redox homeostasis through regulation of ROS generation.

**Fig. S8:**
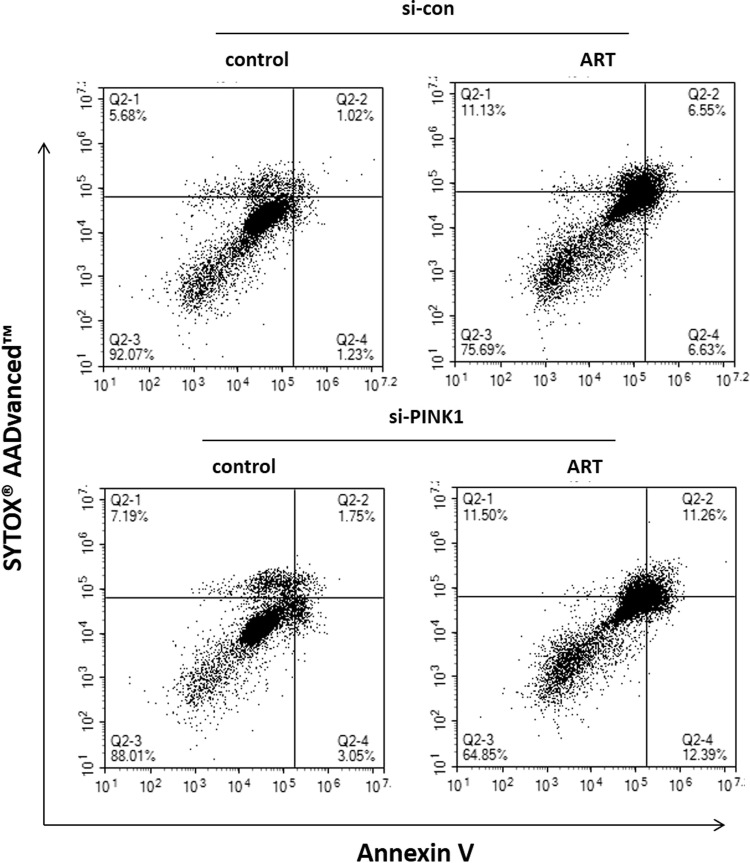
**Suppl Fig. 8. Dual parameter dot plot of HeLa cells stained with apoptosis kit.** HeLa cells were transiently transfected with a nonspecific siRNA or the PINK1-specific siRNA. After 72 h, cells were treated with ART (20 μM) for 24 h and harvested for Pacific Blue™ Annexin V and SYTOX^®^ AADvanced™ co-staining. Flow cytometry was performed to analyze apoptotic cells using 405 nm and 488 nm excitation.

Finally, previous studies [24], [25], [26] have shown that ART causes mROS generation and induces cell apoptosis. We thus examined the effect of PINK1 knockdown on ART-induced cell death. Cell morphology changes showed that PINK1-knockdown cells were more sensitive to ART-caused cell death (Fig. 7**D**). Moreover, we performed Annexin V and SYTOX^®^ AADvanced™ co-staining to quantify cell apoptosis using flow cytometry. As shown in Fig. 7**E** and Suppl Fig. 8, knockdown of PINK1 significantly increased cell apoptosis induced by ART. These findings suggest that mitophagy serves as a cell survival mechanism following ART treatment and that the blockage of mitophagy could sensitize cancer cells to ART-induced apoptosis.

## 4 Discussion

Mitophagy refers to the process of selectively degrading dysfunctional mitochondria [1]. In response to stress stimuli, damaged mitochondrial undergoes fission during apoptosis and mitophagy serves to protect the cells against harm resulting from damaged mitochondria. Here, we showed for the first time that ART induces mitophagy in HeLa cells, which is characterized by the decrease of mitochondrial proteins, the formation of autophagosomes, and its fusion with lysosomes and mitochondria. The molecular mechanism is associated with the activation of the PINK1-dependent pathway and the recruitment of Parkin to mitochondria. We demonstrated that mitophagy is an early response to ART-induced mitochondrial injury that may help to efficiently eliminate damaged mitochondria and maintain cellular homeostasis.

Our previous studies [20] revealed that heme, rather than free ferrous iron, is predominantly responsible for artemisinin activation. Furthermore, we [22] utilized ART -triphenylphosphoniumbromide (ART-TPP) to target artemisinin to the mitochondria where free heme is synthesized. This led to the greatly improved activity of artemisinin. This may be indicative that ART mainly exerts its effect in mitochondria. To confirm this, we utilized LynxTag-ART™ AS_Red_, a red fluorescence-tagged ART, to investigate its cellular localization and observed that ART was predominantly localized in mitochondria (Fig. 1**B**). Moreover, we utilized activity-based protein profiling (ABPP) combining with bio-orthogonal “click” chemistry, which has been widely used for target identification in many natural products, including curcumin [28] and andrographolide [35], to identify ART-binding mitochondrial targets. ABPP not only recapitulates protein-small molecule interactions in situ but also enables the enrichment of these complexes for subsequent large-scale proteome-wide identification of potential targets [36], [37]. As shown in Suppl Table 1 and Fig. 3, a total of 442 proteins were identified as molecular targets of ART, most of which belong to mitochondrial membrane proteins or enzymes for energy metabolism, suggesting that ART alters mitochondrial functions.

Mitophagy is regulated by the PINK1-dependent pathway [1]. PINK1 recruits Parkin from the cytoplasm to damaged mitochondria and subsequently promotes selective degradation of the damaged mitochondria via mitophagy [38]. Mechanistically, we found that the activation of the PINK1-dependent pathway contributes to ART-induced mitophagy. Immunofluorescence (Fig. 5**C**) and immunoprecipitation assays (Fig. 5**D)** showed that ART treatment enhanced the interaction of PINK1 and Parkin. As a result, Parkin was recruited to mitochondria and subsequently ubiquitinate numerous mitochondrial proteins (Fig. 4**D**), which were then recognized by autophagy adaptor SQSTM1 or other molecules interacting with LC3 to form the autophagosomes. Moreover, we utilized the ART-P with an alkyne moiety [20] to reveal the direct interaction of ART with PINK1 or Parkin **(**Fig. 5**E)**. We showed that PINK1 or Parkin are both among the direct molecular targets of ART, constituting an important molecular mechanism of mitophagy induction by ART. With the advances of mass spectrometry technologies, it is feasible to further identify the specific ART-binding sites on protein targets PINK1 or Parkin in future studies. Finally, knockdown of PINK1 reversed the decrease of mitochondrial proteins and decreased the colocalization of autophagosomes, lysosomes and mitochondria by ART (Fig. 6 and Suppl Fig. 4), further confirming the importance of PINK1 in ART-induced mitophagy.

Mitochondria are essential organelles that regulates cellular energy homeostasis and cell death. The removal of damaged mitochondria through mitophagy is thus critical for maintaining proper cellular functions [31], [39]. In our study, we discussed the pathophysiological roles of mitophagy in ART-treated cells. When PINK1 was knocked down in ART-treated cells, the loss of PINK1 resulted in a switch to aerobic glycolysis, a phenomenon similar to the “Warburg effect”, and excessive production of ROS from damaged mitochondria led to dysfunctional oxidative phosphorylation [40]. This is characterized by a significant increase of cellular lactate and mROS levels (Figs. 7**B** and 7**C**). On the contrary, the level of the antioxidant GSH was markedly reduced (Suppl Fig. 7B). Subsequently, the increase in mROS caused oxidative damage to mitochondria and led to more cell apoptosis (Fig. 7**D-E** and Suppl Fig. 8), suggesting the role of mitophagy in cell survival. In addition, knockdown of PINK1 led to more disruption of the mitochondrial membrane potential (Fig. 7**A** and Suppl Fig. 7A), which could a sign of the early stage of cell apoptosis. In the absence of the mitophagy machinery, damaged mitochondria produce cytotoxic ROS, which further damages genomic DNA and disrupts mitochondrial membrane potential, leading to intrinsic mitochondrial apoptosis. The PINK1-dependent mitophagy is therefore protective against the cytotoxic effects of ART, and could be a pathway of interest when considering methods to enhance the efficacy of ART in an anticancer setting.

In conclusion, we have provided evidence to suggest that ART induces mitophagy through the PINK1-dependent pathway, and mitophagy serves as a cell survival mechanism during ART-induced cell apoptosis. ART-induced mitophagy suppresses mitochondrial apoptosis, while knockdown of PINK1 leads to increased mitochondrial apoptosis by ART. Our study describes a mechanism by which the PINK1-dependent pathway mediates the complex balance between mitophagy and apoptosis in HeLa cells. Our findings suggest that mitophagy inhibition, either by inhibitors or interference RNAs specific for PINK1, could be an effective novel strategy to enhance the anticancer activity of ART.
