# Supplementary material for: Artesunate-induced mitophagy alters cellular redox status
Source: Redox Biol. 2018 Aug 4;19:263–73. doi: 10.1016/j.redox.2018.07.025 (PMC6128040; doi:10.1016/j.redox.2018.07.025)
Supplement: Supplementary file 1 — Supplementary material [file mmc9.pdf]

Supplementary table 1. List of ART targets

No.	Unused Score[1]	% Cov (95)[2]	Accession #	Name	Species	Peptides(95%)[3]
1	171.09	28.3	Q14204|DYHC1	Cytoplasmic dynein 1 heavy chain 1	HUMAN	93
2	157.06	50.4	Q13085|ACACA	Acetyl-CoA carboxylase 1	HUMAN	95
3	137.17	43.4	P49327|FAS	Fatty acid synthase	HUMAN	77
4	114.99	57.7	P31327|CPSM	Carbamoyl-phosphate synthase [ammonia], mitochondrial	HUMAN	81
5	113.47	36.5	Q6P2Q9|PRP8	Pre-mRNA-processing-splicing factor 8	HUMAN	63
6	101.29	56.9	P11498|PYC	Pyruvate carboxylase, mitochondrial	HUMAN	82
7	82.54	31.7	P27708|PYR1	CAD protein	HUMAN	45
8	81.76	21.6	Q92616|GCN1	eIF-2-alpha kinase activator GCN1	HUMAN	41
9	78.66	25.7	O75369|FLNB	Filamin-B	HUMAN	46
10	68.85	36.4	Q00610|CLH1	Clathrin heavy chain 1	HUMAN	40
11	59.7	32.5	Q08211|DHX9	ATP-dependent RNA helicase A	HUMAN	31
12	59.29	11.3	Q15149|PLEC	Plectin	HUMAN	28
13	57.25	19.4	O75643|U520	U5 small nuclear ribonucleoprotein 200 kDa helicase	HUMAN	31
14	55.53	31.9	P16615|AT2A2	Sarcoplasmic/endoplasmic reticulum calcium ATPase 2	HUMAN	30
15	50.86	50.5	P49748|ACADV	Very long-chain specific acyl-CoA dehydrogenase, mitochondrial	HUMAN	31
16	50.48	49.5	P40939|ECHA	Trifunctional enzyme subunit alpha, mitochondrial	HUMAN	33
17	46.52	12.5	P15924|DESP	Desmoplakin	HUMAN	25
18	44.91	74.8	P07437|TBB5	Tubulin beta chain	HUMAN	32
19	44.74	36.7	P02786|TFR1	Transferrin receptor protein 1	HUMAN	24
20	42.3	24.1	P41252|SYIC	Isoleucine--tRNA ligase, cytoplasmic	HUMAN	22
21	37.73	25.8	P26640|SYVC	Valine--tRNA ligase	HUMAN	19
22	35.44	26.3	P58107|EPIPL	Epiplakin	HUMAN	21
23	33.06	15.5	Q9NZM1|MYOF	Myoferlin	HUMAN	17
24	31.63	19.2	P42704|LPPRC	Leucine-rich PPR motif-containing protein, mitochondrial	HUMAN	16
25	31.29	62.1	P60709|ACTB	Actin, cytoplasmic 1	HUMAN	23
26	29	20.9	Q7L2E3|DHX30	Putative ATP-dependent RNA helicase DHX30	HUMAN	15
27	28.79	26.7	P08238|HS90B	Heat shock protein HSP 90-beta	HUMAN	14
28	27.97	23.9	P13639|EF2	Elongation factor 2	HUMAN	14
29	27.44	33.4	Q07065|CKAP4	Cytoskeleton-associated protein 4	HUMAN	14
30	27.31	12.4	Q09666|AHNK	Neuroblast differentiation-associated protein AHNAK	HUMAN	15
31	26.4	33.7	P10809|CH60	60 kDa heat shock protein, mitochondrial	HUMAN	16
32	26.18	28.6	P05165|PCCA	Propionyl-CoA carboxylase alpha chain, mitochondrial	HUMAN	14
33	24.45	16.2	Q6UB35|C1TM	Monofunctional C1-tetrahydrofolate synthase, mitochondrial	HUMAN	13
34	24.38	53.8	P30519|HMOX2	Heme oxygenase 2	HUMAN	16
35	24.15	16.4	O00410|IPO5	Importin-5	HUMAN	12
36	23.88	34.1	P14618|KPYM	Pyruvate kinase PKM	HUMAN	13
37	22.91	9.3	Q92621|NU205	Nuclear pore complex protein Nup205	HUMAN	13
38	22.35	14.7	P53396|ACLY	ATP-citrate synthase	HUMAN	11
39	22	23.2	Q92945|FUBP2	Far upstream element-binding protein 2	HUMAN	11
40	21.32	36.1	P34897|GLYM	Serine hydroxymethyltransferase, mitochondrial	HUMAN	12
41	20.21	14.4	P53621|COPA	Coatomer subunit alpha	HUMAN	10
42	20	23.2	P50990|TCPQ	T-complex protein 1 subunit theta	HUMAN	10
43	18	19.9	P02545|LMNA	Prelamin-A/C	HUMAN	9

44	17.89	21.1	Q9BQ52|RNZ2	Zinc phosphodiesterase ELAC protein 2	HUMAN	10
45	17.79	34.1	P00558|PGK1	Phosphoglycerate kinase 1	HUMAN	9
46	17.79	4.4	Q5T4S7|UBR4	E3 ubiquitin-protein ligase UBR4	HUMAN	11
47	17.65	20.7	Q5T9A4|ATD3B	ATPase family AAA domain-containing protein 3B	HUMAN	9
48	17.57	46.4	Q15365|PCBP1	Poly(rC)-binding protein 1	HUMAN	9
49	17	39.1	P63244|RACK1	Receptor of activated protein C kinase 1	HUMAN	9
50	16.65	54.6	Q13162|PRDX4	Peroxiredoxin-4	HUMAN	8
51	16	24.3	Q9Y6M5|ZNT1	Zinc transporter 1	HUMAN	8
52	15.98	44.9	Q9Y6C9|MTCH2	Mitochondrial carrier homolog 2	HUMAN	9
53	15.9	7.8	P35579|MYH9	Myosin-9	HUMAN	9
54	15.31	15.1	P42166|LAP2A	Lamina-associated polypeptide 2, isoform alpha	HUMAN	8
55	15.04	8.3	P07814|SYEP	Bifunctional glutamate/proline--tRNA ligase	HUMAN	7
56	14.6	19.2	Q92841|DDX17	Probable ATP-dependent RNA helicase DDX17	HUMAN	7
57	14.43	35.2	P05141|ADT2	ADP/ATP translocase 2	HUMAN	11
58	14.4	9.2	P49790|NU153	Nuclear pore complex protein Nup153	HUMAN	7
59	14.28	45.8	P09601|HMOX1	Heme oxygenase 1	HUMAN	9
60	14.22	23	P49411|EFTU	Elongation factor Tu, mitochondrial	HUMAN	9
61	14.18	18.9	Q96RQ3|MCCA	Methylcrotonoyl-CoA carboxylase subunit alpha, mitochondrial	HUMAN	7
62	14	15.8	P38646|GRP75	Stress-70 protein, mitochondrial	HUMAN	9
63	14	14.1	Q96RP9|EFGM	Elongation factor G, mitochondrial	HUMAN	7
64	14	22	P36578|RL4	60S ribosomal protein L4	HUMAN	7
65	13.97	16.4	P11142|HSP7C	Heat shock cognate 71 kDa protein	HUMAN	7
66	13.74	17.5	O75844|FACE1	CAAX prenyl protease 1 homolog	HUMAN	8
67	13.64	4.7	P49792|RBP2	E3 SUMO-protein ligase RanBP2	HUMAN	8
68	13.55	12.7	P50416|CPT1A	Carnitine O-palmitoyltransferase 1, liver isoform	HUMAN	8
69	13.07	17.8	P08670|VIME	Vimentin	HUMAN	7
70	12.53	9.4	P05023|AT1A1	Sodium/potassium-transporting ATPase subunit alpha-1	HUMAN	7
71	12.44	13.2	P54886|P5CS	Delta-1-pyrroline-5-carboxylate synthase	HUMAN	6
72	12.35	21.1	P07237|PDIA1	Protein disulfide-isomerase	HUMAN	7
73	12.28	33.7	P04406|G3P	Glyceraldehyde-3-phosphate dehydrogenase	HUMAN	7
74	12	14.4	P36776|LONM	Lon protease homolog, mitochondrial	HUMAN	6
75	12	18.4	P41091|IF2G	Eukaryotic translation initiation factor 2 subunit 3	HUMAN	8
76	12	49.1	P62937|PPIA	Peptidyl-prolyl cis-trans isomerase A	HUMAN	6
77	12	25.3	Q32P51|RA1L2	Heterogeneous nuclear ribonucleoprotein A1-like 2	HUMAN	6
78	11.94	22.5	Q9H857|NT5D2	5'-nucleotidase domain-containing protein 2	HUMAN	6
79	11.9	10.1	O95373|IPO7	Importin-7	HUMAN	7
80	11.64	25.6	P06733|ENOA	Alpha-enolase	HUMAN	7
81	11.46	17.4	P78371|TCPB	T-complex protein 1 subunit beta	HUMAN	6
82	11.45	38.7	Q06830|PRDX1	Peroxiredoxin-1	HUMAN	6
83	11.36	5.2	Q12789|TF3C1	General transcription factor 3C polypeptide 1	HUMAN	6
84	10.98	13.5	P28288|ABCD3	ATP-binding cassette sub-family D member 3	HUMAN	6
85	10.94	26.9	P45880|VDAC2	Voltage-dependent anion-selective channel protein 2	HUMAN	6
86	10.88	19.3	P29401|TKT	Transketolase	HUMAN	5
87	10.77	5.2	P46013|KI67	Proliferation marker protein Ki-67	HUMAN	6
88	10.67	16	Q16881|TRXR1	Thioredoxin reductase 1, cytoplasmic	HUMAN	5
89	10.64	7.3	O60306|AQR	Intron-binding protein aquarius	HUMAN	6
90	10.6	17.1	Q00325|MPCP	Phosphate carrier protein, mitochondrial	HUMAN	7
91	10.28	13.5	P08195|4F2	4F2 cell-surface antigen heavy chain	HUMAN	6
92	10.23	25.4	P53701|CCHL	Cytochrome c-type heme lyase	HUMAN	6
93	10.09	10	P43304|GPDM	Glycerol-3-phosphate dehydrogenase, mitochondrial	HUMAN	5

94	10.05	9.2	O94901|SUN1	SUN domain-containing protein 1	HUMAN	6
95	10	10.2	P11021|GRP78	78 kDa glucose-regulated protein	HUMAN	5
96	10	14.3	O43175|SERA	D-3-phosphoglycerate dehydrogenase	HUMAN	5
97	10	14.8	P23526|SAHH	Adenosylhomocysteinase	HUMAN	5
98	10	11.4	Q00839|HNRPU	Heterogeneous nuclear ribonucleoprotein U	HUMAN	8
99	9.82	10.4	P46977|STT3A	Dolichyl-diphosphooligosaccharide--protein glycosyltransferase subu	HUMAN	5
100	9.69	13.1	P30101|PDIA3	Protein disulfide-isomerase A3	HUMAN	5
101	9.61	7.8	P55265|DSRAD	Double-stranded RNA-specific adenosine deaminase	HUMAN	5
102	9.54	9	O00411|RPOM	DNA-directed RNA polymerase, mitochondrial	HUMAN	6
103	9.49	2.2	O75691|UTP20	Small subunit processome component 20 homolog	HUMAN	5
104	8.85	29.7	P30048|PRDX3	Thioredoxin-dependent peroxide reductase, mitochondrial	HUMAN	6
105	8.67	9.9	Q13200|PSMD2	26S proteasome non-ATPase regulatory subunit 2	HUMAN	4
106	8.49	13.4	Q9H845|ACAD9	Acyl-CoA dehydrogenase family member 9, mitochondrial	HUMAN	5
107	8.2	5.3	Q14997|PSME4	Proteasome activator complex subunit 4	HUMAN	4
108	8.16	18.7	Q9H5Q4|TFB2M	Dimethyladenosine transferase 2, mitochondrial	HUMAN	6
109	8.13	16.2	Q8TB36|GDAP1	Ganglioside-induced differentiation-associated protein 1	HUMAN	4
110	8.13	50.7	O14880|MGST3	Microsomal glutathione S-transferase 3	HUMAN	5
111	8.08	16	P00338|LDHA	L-lactate dehydrogenase A chain	HUMAN	4
112	8.06	15	O00571|DDX3X	ATP-dependent RNA helicase DDX3X	HUMAN	4
113	8.01	6.6	O43707|ACTN4	Alpha-actinin-4	HUMAN	4
114	8	8.5	Q12931|TRAP1	Heat shock protein 75 kDa, mitochondrial	HUMAN	5
115	8	13.2	P12268|IMDH2	Inosine-5'-monophosphate dehydrogenase 2	HUMAN	4
116	8	24.4	Q02543|RL18A	60S ribosomal protein L18a	HUMAN	4
117	7.9	7.3	O75694|NU155	Nuclear pore complex protein Nup155	HUMAN	5
118	7.83	7.9	Q01813|PFKAP	ATP-dependent 6-phosphofructokinase, platelet type	HUMAN	4
119	7.78	10.9	P48643|TCPE	T-complex protein 1 subunit epsilon	HUMAN	4
120	7.68	22.1	P31943|HNRH1	Heterogeneous nuclear ribonucleoprotein H	HUMAN	4
121	7.68	3.8	Q8TEQ6|GEMI5	Gem-associated protein 5	HUMAN	4
122	7.57	13.8	P60842|IF4A1	Eukaryotic initiation factor 4A-I	HUMAN	4
123	7.31	8.3	P10515|ODP2	Dihydrolipoyllysine-residue acetyltransferase component of pyruvate	HUMAN	4
124	7.2	13.7	Q9BZE1|RM37	39S ribosomal protein L37, mitochondrial	HUMAN	4
125	7.03	6.5	Q15393|SF3B3	Splicing factor 3B subunit 3	HUMAN	3
126	7.01	10.3	P03915|NU5M	NADH-ubiquinone oxidoreductase chain 5	HUMAN	4
127	6.79	15	P07195|LDHB	L-lactate dehydrogenase B chain	HUMAN	4
128	6.59	12.5	P61978|HNRPK	Heterogeneous nuclear ribonucleoprotein K	HUMAN	3
129	6.51	17.9	P30041|PRDX6	Peroxiredoxin-6	HUMAN	4
130	6.49	13.4	Q5TFE4|NT5D1	5'-nucleotidase domain-containing protein 1	HUMAN	4
131	6.46	27.7	P18621|RL17	60S ribosomal protein L17	HUMAN	4
132	6.41	15	P13196|HEM1	5-aminolevulinate synthase, nonspecific, mitochondrial	HUMAN	4
133	6.4	11.1	P31040|SDHA	Succinate dehydrogenase [ubiquinone] flavoprotein subunit, mitocho	HUMAN	3
134	6.39	11.8	Q92552|RT27	28S ribosomal protein S27, mitochondrial	HUMAN	3
135	6.33	13.3	O14832|PAHX	Phytanoyl-CoA dioxygenase, peroxisomal	HUMAN	4
136	6.33	3.4	Q93008|USP9X	Probable ubiquitin carboxyl-terminal hydrolase FAF-X	HUMAN	4
137	6.24	14.1	Q9H7Z7|PGES2	Prostaglandin E synthase 2	HUMAN	4
138	6.23	14.7	O75616|ERAL1	GTPase Era, mitochondrial	HUMAN	3
139	6.08	20.6	P04075|ALDOA	Fructose-bisphosphate aldolase A	HUMAN	3
140	6.06	12.8	P26641|EF1G	Elongation factor 1-gamma	HUMAN	3
141	6.05	4.9	Q86VP6|CAND1	Cullin-associated NEDD8-dissociated protein 1	HUMAN	3
142	6.05	19.4	Q9Y277|VDAC3	Voltage-dependent anion-selective channel protein 3	HUMAN	3
143	6.04	5.2	P56192|SYMC	Methionine--tRNA ligase, cytoplasmic	HUMAN	3

144	6.03	17.4	Q07021|C1QBP	Complement component 1 Q subcomponent-binding protein, mitoch	HUMAN	3
145	6.02	14.1	Q9Y2S7|PDIP2	Polymerase delta-interacting protein 2	HUMAN	3
146	6	44.5	O43169|CYB5B	Cytochrome b5 type B	HUMAN	5
147	6	6.8	P05091|ALDH2	Aldehyde dehydrogenase, mitochondrial	HUMAN	3
148	6	6.4	P53985|MOT1	Monocarboxylate transporter 1	HUMAN	3
149	6	15.7	P62753|RS6	40S ribosomal protein S6	HUMAN	3
150	6	67.4	P68371|TBB4B	Tubulin beta-4B chain	HUMAN	28
151	6	31.4	P07737|PROF1	Profilin-1	HUMAN	3
152	6	16.2	P61313|RL15	60S ribosomal protein L15	HUMAN	3
153	6	8.9	Q15084|PDIA6	Protein disulfide-isomerase A6	HUMAN	3
154	5.96	6.4	P42892|ECE1	Endothelin-converting enzyme 1	HUMAN	3
155	5.91	8.3	Q14974|IMB1	Importin subunit beta-1	HUMAN	4
156	5.89	4.2	Q5ST30|SYVM	Valine--tRNA ligase, mitochondrial	HUMAN	3
157	5.86	3.9	Q14980|NUMA1	Nuclear mitotic apparatus protein 1	HUMAN	3
158	5.85	7	Q13838|DX39B	Spliceosome RNA helicase DDX39B	HUMAN	3
159	5.81	11.7	P49368|TCPG	T-complex protein 1 subunit gamma	HUMAN	3
160	5.6	5.5	Q9UHI6|DDX20	Probable ATP-dependent RNA helicase DDX20	HUMAN	3
161	5.59	9.3	Q9BT22|ALG1	Chitobiosyldiphosphodolichol beta-mannosyltransferase	HUMAN	3
162	5.55	7	Q16822|PCKGM	Phosphoenolpyruvate carboxykinase [GTP], mitochondrial	HUMAN	3
163	5.46	9.1	Q16850|CP51A	Lanosterol 14-alpha demethylase	HUMAN	3
164	5.39	28.5	P27635|RL10	60S ribosomal protein L10	HUMAN	6
165	5.37	22.3	P46776|RL27A	60S ribosomal protein L27a	HUMAN	3
166	5.28	7	P06576|ATPB	ATP synthase subunit beta, mitochondrial	HUMAN	3
167	5.25	8.8	P14625|ENPL	Endoplasmin	HUMAN	4
168	5.24	11.9	P31930|QCR1	Cytochrome b-c1 complex subunit 1, mitochondrial	HUMAN	3
169	5.18	12.8	P36542|ATPG	ATP synthase subunit gamma, mitochondrial	HUMAN	3
170	5.13	13.1	P51398|RT29	28S ribosomal protein S29, mitochondrial	HUMAN	4
171	5.11	12.6	P14866|HNRPL	Heterogeneous nuclear ribonucleoprotein L	HUMAN	3
172	5.07	3.3	Q9NQC3|RTN4	Reticulon-4	HUMAN	3
173	5.02	18.3	P07355|ANXA2	Annexin A2	HUMAN	3
174	4.98	2	O95714|HERC2	E3 ubiquitin-protein ligase HERC2	HUMAN	3
175	4.89	4.5	P11388|TOP2A	DNA topoisomerase 2-alpha	HUMAN	3
176	4.85	38.1	Q71UM5|RS27L	40S ribosomal protein S27-like	HUMAN	3
177	4.81	11	P21796|VDAC1	Voltage-dependent anion-selective channel protein 1	HUMAN	3
178	4.62	34.8	Q9BUF5|TBB6	Tubulin beta-6 chain	HUMAN	15
179	4.57	7.5	Q9UHG3|PCYOX	Prenylcysteine oxidase 1	HUMAN	3
180	4.56	16.5	Q99714|HCD2	3-hydroxyacyl-CoA dehydrogenase type-2	HUMAN	2
181	4.53	7.5	Q16891|MIC60	MICOS complex subunit MIC60	HUMAN	3
182	4.5	15.3	Q8TED1|GPX8	Probable glutathione peroxidase 8	HUMAN	3
183	4.46	5.3	Q9BW92|SYTM	Threonine--tRNA ligase, mitochondrial	HUMAN	3
184	4.46	34.3	P23528|COF1	Cofilin-1	HUMAN	2
185	4.4	18	Q8NHW5|RLA0L	60S acidic ribosomal protein P0-like	HUMAN	2
186	4.38	12.6	P08574|CY1	Cytochrome c1, heme protein, mitochondrial	HUMAN	3
187	4.37	13.5	Q9NX40|OCAD1	OCIA domain-containing protein 1	HUMAN	3
188	4.35	1.4	Q9Y4A5|TRRAP	Transformation/transcription domain-associated protein	HUMAN	2
189	4.34	7.6	Q13310|PABP4	Polyadenylate-binding protein 4	HUMAN	2
190	4.3	5.3	Q9BUJ2|HNRL1	Heterogeneous nuclear ribonucleoprotein U-like protein 1	HUMAN	2
191	4.27	15.9	P52597|HNRPF	Heterogeneous nuclear ribonucleoprotein F	HUMAN	4
192	4.22	15	Q9UMS0|NFU1	NFU1 iron-sulfur cluster scaffold homolog, mitochondrial	HUMAN	2
193	4.16	30.5	P63241|IF5A1	Eukaryotic translation initiation factor 5A-1	HUMAN	2

194	4.14	14.8	P17844|DDX5	Probable ATP-dependent RNA helicase DDX5	HUMAN	4
195	4.13	9.6	P55072|TERA	Transitional endoplasmic reticulum ATPase	HUMAN	3
196	4.11	2.1	Q8N3C0|ASCC3	Activating signal cointegrator 1 complex subunit 3	HUMAN	2
197	4.1	8.8	Q6PIU2|NCEH1	Neutral cholesterol ester hydrolase 1	HUMAN	2
198	4.08	15.1	P08559|ODPA	Pyruvate dehydrogenase E1 component subunit alpha, somatic form,	HUMAN	3
199	4.08	7.6	Q14566|MCM6	DNA replication licensing factor MCM6	HUMAN	3
200	4.07	7.5	P26639|SYTC	Threonine--tRNA ligase, cytoplasmic	HUMAN	2
201	4.02	6.5	Q9P258|RCC2	Protein RCC2	HUMAN	2
202	4.01	7.2	P00367|DHE3	Glutamate dehydrogenase 1, mitochondrial	HUMAN	2
203	4.01	6.2	Q969Z0|TBRG4	Protein TBRG4	HUMAN	2
204	4.01	13.9	P46781|RS9	40S ribosomal protein S9	HUMAN	2
205	4	22.9	P10599|THIO	Thioredoxin	HUMAN	2
206	4	9.1	P32322|P5CR1	Pyrroline-5-carboxylate reductase 1, mitochondrial	HUMAN	2
207	4	4.4	P34932|HSP74	Heat shock 70 kDa protein 4	HUMAN	2
208	4	3.1	Q12906|ILF3	Interleukin enhancer-binding factor 3	HUMAN	2
209	4	4.1	Q5JRX3|PREP	Presequence protease, mitochondrial	HUMAN	2
210	4	44.8	Q96IX5|USMG5	Up-regulated during skeletal muscle growth protein 5	HUMAN	2
211	4	1.5	Q9H583|HEAT1	HEAT repeat-containing protein 1	HUMAN	2
212	4	15.3	O15258|RER1	Protein RER1	HUMAN	2
213	4	2.2	O95197|RTN3	Reticulon-3	HUMAN	2
214	4	33.7	P04080|CYTB	Cystatin-B	HUMAN	2
215	4	12.7	P04792|HSPB1	Heat shock protein beta-1	HUMAN	2
216	4	10.7	P21266|GSTM3	Glutathione S-transferase Mu 3	HUMAN	2
217	4	11.3	P40429|RL13A	60S ribosomal protein L13a	HUMAN	2
218	4	5.2	P43243|MATR3	Matrin-3	HUMAN	2
219	4	9.8	P46777|RL5	60S ribosomal protein L5	HUMAN	2
220	4	12.9	P62913|RL11	60S ribosomal protein L11	HUMAN	2
221	4	32.9	P63173|RL38	60S ribosomal protein L38	HUMAN	2
222	4	28.2	P69905|HBA	Hemoglobin subunit alpha	HUMAN	2
223	4	9.2	P84098|RL19	60S ribosomal protein L19	HUMAN	2
224	4	13.8	Q07020|RL18	60S ribosomal protein L18	HUMAN	2
225	4	15.9	Q15366|PCBP2	Poly(rC)-binding protein 2	HUMAN	4
226	4	6.1	Q16658|FSCN1	Fascin	HUMAN	2
227	4	15.9	Q6UW68|TM205	Transmembrane protein 205	HUMAN	2
228	4	48.6	Q9BQE3|TBA1C	Tubulin alpha-1C chain	HUMAN	17
229	4	3.2	Q9NZB2|F120A	Constitutive coactivator of PPAR-gamma-like protein 1	HUMAN	2
230	3.96	3.7	Q03518|TAP1	Antigen peptide transporter 1	HUMAN	2
231	3.96	3.7	Q16678|CP1B1	Cytochrome P450 1B1	HUMAN	2
232	3.96	5.6	Q96GW9|SYMM	Methionine--tRNA ligase, mitochondrial	HUMAN	2
233	3.93	4	Q02218|ODO1	2-oxoglutarate dehydrogenase, mitochondrial	HUMAN	2
234	3.92	4.6	Q92685|ALG3	Dol-P-Man:Man(5)GlcNAc(2)-PP-Dol alpha-1,3-mannosyltransferase	HUMAN	2
235	3.9	5.9	Q99832|TCPH	T-complex protein 1 subunit eta	HUMAN	2
236	3.89	9.4	Q9NYP7|ELOV5	Elongation of very long chain fatty acids protein 5	HUMAN	2
237	3.85	20.3	Q5RI15|COX20	Cytochrome c oxidase protein 20 homolog	HUMAN	2
238	3.84	25.7	P15531|NDKA	Nucleoside diphosphate kinase A	HUMAN	3
239	3.74	17.4	P25398|RS12	40S ribosomal protein S12	HUMAN	3
240	3.73	7.5	Q9NSE4|SYIM	Isoleucine--tRNA ligase, mitochondrial	HUMAN	2
241	3.66	13.7	P47756|CAPZB	F-actin-capping protein subunit beta	HUMAN	2
242	3.64	14.6	P60900|PSA6	Proteasome subunit alpha type-6	HUMAN	2
243	3.6	11.6	P22626|ROA2	Heterogeneous nuclear ribonucleoproteins A2/B1	HUMAN	3

244	3.6	3.1	Q9BQG0|MBB1A	Myb-binding protein 1A	HUMAN	2
245	3.55	6.6	P52292|IMA1	Importin subunit alpha-1	HUMAN	3
246	3.5	8.4	O14980|XPO1	Exportin-1	HUMAN	2
247	3.48	4.4	P55084|ECHB	Trifunctional enzyme subunit beta, mitochondrial	HUMAN	2
248	3.44	4.5	O75027|ABCB7	ATP-binding cassette sub-family B member 7, mitochondrial	HUMAN	2
249	3.42	1.5	P35573|GDE	Glycogen debranching enzyme	HUMAN	2
250	3.38	15	Q86YH6|DLP1	Decaprenyl-diphosphate synthase subunit 2	HUMAN	2
251	3.34	2.3	A5YKK6|CNOT1	CCR4-NOT transcription complex subunit 1	HUMAN	2
252	3.28	5.8	Q7KZF4|SND1	Staphylococcal nuclease domain-containing protein 1	HUMAN	2
253	3.27	10.3	P09001|RM03	39S ribosomal protein L3, mitochondrial	HUMAN	2
254	3.21	13.3	Q9H2D1|MFTC	Mitochondrial folate transporter/carrier	HUMAN	2
255	3.19	51.3	P62888|RL30	60S ribosomal protein L30	HUMAN	2
256	3.17	3.1	Q92598|HS105	Heat shock protein 105 kDa	HUMAN	2
257	3.13	4.1	P31948|STIP1	Stress-induced-phosphoprotein 1	HUMAN	2
258	3.12	14.4	P62341|SELT	Selenoprotein T	HUMAN	2
259	3.1	0.8	Q9NU22|MDN1	Midasin	HUMAN	2
260	3.09	6.2	Q3ZCQ8|TIM50	Mitochondrial import inner membrane translocase subunit TIM50	HUMAN	2
261	3.08	4.9	P07339|CATD	Cathepsin D	HUMAN	2
262	3.08	12.9	Q02878|RL6	60S ribosomal protein L6	HUMAN	3
263	3.07	3.9	O15091|MRRP3	Mitochondrial ribonuclease P protein 3	HUMAN	2
264	3.05	4.9	Q16576|RBBP7	Histone-binding protein RBBP7	HUMAN	2
265	3.04	5.1	P09874|PARP1	Poly [ADP-ribose] polymerase 1	HUMAN	2
266	3.04	7.6	P25705|ATPA	ATP synthase subunit alpha, mitochondrial	HUMAN	2
267	3.04	6.4	P53007|TXTP	Tricarboxylate transport protein, mitochondrial	HUMAN	2
268	3.01	4.3	P30876|RPB2	DNA-directed RNA polymerase II subunit RPB2	HUMAN	2
269	2.97	10.2	P08243|ASNS	Asparagine synthetase [glutamine-hydrolyzing]	HUMAN	2
270	2.97	7.2	Q9H7H0|MET17	Methyltransferase-like protein 17, mitochondrial	HUMAN	2
271	2.97	4	O43143|DHX15	Pre-mRNA-splicing factor ATP-dependent RNA helicase DHX15	HUMAN	1
272	2.96	5.8	P00505|AATM	Aspartate aminotransferase, mitochondrial	HUMAN	2
273	2.95	11.1	P42765|THIM	3-ketoacyl-CoA thiolase, mitochondrial	HUMAN	2
274	2.93	2.4	P11586|C1TC	C-1-tetrahydrofolate synthase, cytoplasmic	HUMAN	2
275	2.93	6.9	Q9BXW7|CECR5	Cat eye syndrome critical region protein 5	HUMAN	2
276	2.89	19.1	P83731|RL24	60S ribosomal protein L24	HUMAN	2
277	2.89	4.5	P13010|XRCC5	X-ray repair cross-complementing protein 5	HUMAN	2
278	2.89	1.7	Q14643|ITPR1	Inositol 1,4,5-trisphosphate receptor type 1	HUMAN	1
279	2.87	3.3	P22314|UBA1	Ubiquitin-like modifier-activating enzyme 1	HUMAN	2
280	2.79	5.1	P17812|PYRG1	CTP synthase 1	HUMAN	2
281	2.78	7.2	P55209|NP1L1	Nucleosome assembly protein 1-like 1	HUMAN	2
282	2.76	24.6	P62244|RS15A	40S ribosomal protein S15a	HUMAN	1
283	2.62	0.9	Q7Z6Z7|HUWE1	E3 ubiquitin-protein ligase HUWE1	HUMAN	1
284	2.61	6.9	P50991|TCPD	T-complex protein 1 subunit delta	HUMAN	2
285	2.59	8.2	Q9NZJ7|MTCH1	Mitochondrial carrier homolog 1	HUMAN	1
286	2.59	2.1	P52701|MSH6	DNA mismatch repair protein Msh6	HUMAN	1
287	2.56	7.4	P17987|TCPA	T-complex protein 1 subunit alpha	HUMAN	2
288	2.52	7.7	Q96SZ6|CK5P1	CDK5 regulatory subunit-associated protein 1	HUMAN	1
289	2.48	6	P40227|TCPZ	T-complex protein 1 subunit zeta	HUMAN	2
290	2.48	4.4	Q93009|UBP7	Ubiquitin carboxyl-terminal hydrolase 7	HUMAN	1
291	2.48	3.7	Q96I24|FUBP3	Far upstream element-binding protein 3	HUMAN	2
292	2.45	8.7	P0DMV9|HS71B	Heat shock 70 kDa protein 1B	HUMAN	3
293	2.43	2.7	Q9HAV4|XPO5	Exportin-5	HUMAN	1

294	2.42	4.6	Q9H0A0|NAT10	RNA cytidine acetyltransferase	HUMAN	1
295	2.41	4	Q13263|TIF1B	Transcription intermediary factor 1-beta	HUMAN	1
296	2.36	7.6	Q99536|VAT1	Synaptic vesicle membrane protein VAT-1 homolog	HUMAN	2
297	2.33	2.7	P27816|MAP4	Microtubule-associated protein 4	HUMAN	2
298	2.31	4.4	Q8NBS9|TXND5	Thioredoxin domain-containing protein 5	HUMAN	1
299	2.3	5.4	Q5JTH9|RRP12	RRP12-like protein	HUMAN	1
300	2.29	7.5	P52272|HNRPM	Heterogeneous nuclear ribonucleoprotein M	HUMAN	1
301	2.29	1.5	Q9ULT8|HECD1	E3 ubiquitin-protein ligase HECTD1	HUMAN	1
302	2.28	4.7	Q96TA2|YMEL1	ATP-dependent zinc metalloprotease YME1L1	HUMAN	1
303	2.28	4.4	O15371|EIF3D	Eukaryotic translation initiation factor 3 subunit D	HUMAN	1
304	2.26	7.5	Q96AG4|LRC59	Leucine-rich repeat-containing protein 59	HUMAN	1
305	2.26	3.9	Q96EY7|PTCD3	Pentatricopeptide repeat domain-containing protein 3, mitochondria	HUMAN	1
306	2.23	7	Q9ULV4|COR1C	Coronin-1C	HUMAN	1
307	2.15	5	Q9Y305|ACOT9	Acyl-coenzyme A thioesterase 9, mitochondrial	HUMAN	1
308	2.14	4.6	Q99797|MIPEP	Mitochondrial intermediate peptidase	HUMAN	1
309	2.13	8.1	P28331|NDUS1	NADH-ubiquinone oxidoreductase 75 kDa subunit, mitochondrial	HUMAN	1
310	2.12	13	P62249|RS16	40S ribosomal protein S16	HUMAN	2
311	2.09	7.1	P08754|GNAI3	Guanine nucleotide-binding protein G(k) subunit alpha	HUMAN	1
312	2.09	10.2	P09493|TPM1	Tropomyosin alpha-1 chain	HUMAN	1
313	2.07	9.7	P62826|RAN	GTP-binding nuclear protein Ran	HUMAN	1
314	2.07	4.2	P35610|SOAT1	Sterol O-acyltransferase 1	HUMAN	1
315	2.06	4.8	O60506|HNRPQ	Heterogeneous nuclear ribonucleoprotein Q	HUMAN	1
316	2.05	4	P13667|PDIA4	Protein disulfide-isomerase A4	HUMAN	1
317	2.03	21.9	P62987|RL40	Ubiquitin-60S ribosomal protein L40	HUMAN	2
318	2.02	10.2	P24539|AT5F1	ATP synthase F(0) complex subunit B1, mitochondrial	HUMAN	2
319	2.02	4.3	P47897|SYQ	Glutamine--tRNA ligase	HUMAN	1
320	2.02	27	Q14061|COX17	Cytochrome c oxidase copper chaperone	HUMAN	3
321	2.02	2.8	O94906|PRP6	Pre-mRNA-processing factor 6	HUMAN	1
322	2.02	4.5	Q15392|DHC24	Delta(24)-sterol reductase	HUMAN	1
323	2.02	3.4	Q9BSJ5|CQ080	Uncharacterized protein C17orf80	HUMAN	1
324	2	4.2	O00170|AIP	AH receptor-interacting protein	HUMAN	1
325	2	4.3	O00217|NDUS8	NADH dehydrogenase [ubiquinone] iron-sulfur protein 8, mitochondr	HUMAN	1
326	2	12.4	O00483|NDUA4	Cytochrome c oxidase subunit NDUFA4	HUMAN	1
327	2	4.3	O75439|MPPB	Mitochondrial-processing peptidase subunit beta	HUMAN	1
328	2	3.3	O75600|KBL	2-amino-3-ketobutyrate coenzyme A ligase, mitochondrial	HUMAN	1
329	2	6.1	O75608|LYPA1	Acyl-protein thioesterase 1	HUMAN	1
330	2	3.1	O94925|GLSK	Glutaminase kidney isoform, mitochondrial	HUMAN	1
331	2	6.9	P12081|SYHC	Histidine--tRNA ligase, cytoplasmic	HUMAN	1
332	2	4.5	P18754|RCC1	Regulator of chromosome condensation	HUMAN	1
333	2	3.1	P19367|HXK1	Hexokinase-1	HUMAN	1
334	2	2	P27824|CALX	Calnexin	HUMAN	1
335	2	2.7	P30837|AL1B1	Aldehyde dehydrogenase X, mitochondrial	HUMAN	1
336	2	3	P41250|SYG	Glycine--tRNA ligase	HUMAN	1
337	2	4.5	P49821|NDUV1	NADH dehydrogenase [ubiquinone] flavoprotein 1, mitochondrial	HUMAN	1
338	2	4.6	P53597|SUCA	Succinate--CoA ligase [ADP/GDP-forming] subunit alpha, mitochondri	HUMAN	1
339	2	13.7	P61604|CH10	10 kDa heat shock protein, mitochondrial	HUMAN	1
340	2	8.6	P62263|RS14	40S ribosomal protein S14	HUMAN	1
341	2	24.7	P63167|DYL1	Dynein light chain 1, cytoplasmic	HUMAN	1
342	2	41.1	P68366|TBA4A	Tubulin alpha-4A chain	HUMAN	15
343	2	1.4	Q08J23|NSUN2	tRNA (cytosine(34)-C(5))-methyltransferase	HUMAN	1

344	2	1.2	Q13423|NNTM	NAD(P) transhydrogenase, mitochondrial	HUMAN	1
345	2	2.7	Q3SXM5|HSDL1	Inactive hydroxysteroid dehydrogenase-like protein 1	HUMAN	1
346	2	5.8	Q567V2|M17L2	Mpv17-like protein 2	HUMAN	1
347	2	2.6	Q7Z434|MAVS	Mitochondrial antiviral-signaling protein	HUMAN	1
348	2	16.9	Q8N4Q1|MIA40	Mitochondrial intermembrane space import and assembly protein 40	HUMAN	1
349	2	6	Q96TC7|RMD3	Regulator of microtubule dynamics protein 3	HUMAN	1
350	2	7.4	Q9BYC9|RM20	39S ribosomal protein L20, mitochondrial	HUMAN	1
351	2	3.5	Q9NP81|SYSM	Serine--tRNA ligase, mitochondrial	HUMAN	1
352	2	2.6	Q9NVV4|PAPD1	Poly(A) RNA polymerase, mitochondrial	HUMAN	1
353	2	3.9	Q9P035|HACD3	Very-long-chain (3R)-3-hydroxyacyl-CoA dehydratase 3	HUMAN	1
354	2	3.1	O00541|PESC	Pescadillo homolog	HUMAN	1
355	2	4.6	O14828|SCAM3	Secretory carrier-associated membrane protein 3	HUMAN	1
356	2	4.8	O15260|SURF4	Surfeit locus protein 4	HUMAN	1
357	2	6.2	O60831|PRAF2	PRA1 family protein 2	HUMAN	1
358	2	2.2	O94919|ENDD1	Endonuclease domain-containing 1 protein	HUMAN	1
359	2	2.4	P00734|THRB	Prothrombin	HUMAN	1
360	2	0.2	P04114|APOB	Apolipoprotein B-100	HUMAN	1
361	2	3.6	P07910|HNRPC	Heterogeneous nuclear ribonucleoproteins C1/C2	HUMAN	1
362	2	8.1	P08708|RS17	40S ribosomal protein S17	HUMAN	1
363	2	3.8	P15880|RS2	40S ribosomal protein S2	HUMAN	1
364	2	0.8	P21359|NF1	Neurofibromin	HUMAN	1
365	2	4.3	P26373|RL13	60S ribosomal protein L13	HUMAN	1
366	2	8.5	P29692|EF1D	Elongation factor 1-delta	HUMAN	1
367	2	9.1	P30050|RL12	60S ribosomal protein L12	HUMAN	1
368	2	10.2	P35268|RL22	60S ribosomal protein L22	HUMAN	1
369	2	9.4	P46778|RL21	60S ribosomal protein L21	HUMAN	1
370	2	2.7	P48651|PTSS1	Phosphatidylserine synthase 1	HUMAN	1
371	2	6.3	P50395|GDIB	Rab GDP dissociation inhibitor beta	HUMAN	1
372	2	5.6	P50914|RL14	60S ribosomal protein L14	HUMAN	1
373	2	12.7	P55795|HNRH2	Heterogeneous nuclear ribonucleoprotein H2	HUMAN	3
374	2	4.9	P57088|TMM33	Transmembrane protein 33	HUMAN	1
375	2	5.3	P60891|PRPS1	Ribose-phosphate pyrophosphokinase 1	HUMAN	1
376	2	5.3	P61158|ARP3	Actin-related protein 3	HUMAN	1
377	2	7.7	P62266|RS23	40S ribosomal protein S23	HUMAN	1
378	2	13	P62854|RS26	40S ribosomal protein S26	HUMAN	1
379	2	15.7	P68871|HBB	Hemoglobin subunit beta	HUMAN	1
380	2	12.1	Q01629|IFM2	Interferon-induced transmembrane protein 2	HUMAN	2
381	2	3.6	Q01650|LAT1	Large neutral amino acids transporter small subunit 1	HUMAN	1
382	2	1.9	Q10471|GALT2	Polypeptide N-acetylgalactosaminyltransferase 2	HUMAN	1
383	2	8.3	Q10589|BST2	Bone marrow stromal antigen 2	HUMAN	1
384	2	2.6	Q12905|ILF2	Interleukin enhancer-binding factor 2	HUMAN	1
385	2	2.8	Q14684|RRP1B	Ribosomal RNA processing protein 1 homolog B	HUMAN	1
386	2	2	Q5SNT2|TM201	Transmembrane protein 201	HUMAN	1
387	2	3	Q6NUM9|RETST	All-trans-retinol 13,14-reductase	HUMAN	1
388	2	5.4	Q6NUT3|MFS12	Major facilitator superfamily domain-containing protein 12	HUMAN	1
389	2	1.4	Q7Z2K6|ERMP1	Endoplasmic reticulum metallopeptidase 1	HUMAN	1
390	2	1.8	Q8IYD1|ERF3B	Eukaryotic peptide chain release factor GTP-binding subunit ERF3B	HUMAN	1
391	2	2.3	Q8IZV5|RDH10	Retinol dehydrogenase 10	HUMAN	1
392	2	3.6	Q8NFJ5|RAI3	Retinoic acid-induced protein 3	HUMAN	1
393	2	2.2	Q8WTV0|SCRB1	Scavenger receptor class B member 1	HUMAN	1

394	2	2.5	Q92769|HDAC2	Histone deacetylase 2	HUMAN	1
395	2	9.9	Q96AX2|RAB37	Ras-related protein Rab-37	HUMAN	1
396	2	3.6	Q96N66|MBOA7	Lysophospholipid acyltransferase 7	HUMAN	1
397	2	3	Q99729|ROAA	Heterogeneous nuclear ribonucleoprotein A/B	HUMAN	1
398	2	3.3	Q9BWF3|RBM4	RNA-binding protein 4	HUMAN	1
399	2	2.3	Q9P2J5|SYLC	Leucine--tRNA ligase, cytoplasmic	HUMAN	1
400	2	4.3	Q9UQ80|PA2G4	Proliferation-associated protein 2G4	HUMAN	1
401	2	8.4	Q9Y3E5|PTH2	Peptidyl-tRNA hydrolase 2, mitochondrial	HUMAN	1
402	2	4.6	Q9Y3F4|STRAP	Serine-threonine kinase receptor-associated protein	HUMAN	1
403	2	8.5	Q9Y676|RT18B	28S ribosomal protein S18b, mitochondrial	HUMAN	1
404	2	4.3	Q9Y6K0|CEPT1	Choline/ethanolaminephosphotransferase 1	HUMAN	1
405	1.96	2.5	Q7L0Y3|MRRP1	Mitochondrial ribonuclease P protein 1	HUMAN	1
406	1.96	1.8	P49588|SYAC	Alanine--tRNA ligase, cytoplasmic	HUMAN	1
407	1.94	7.2	Q99873|ANM1	Protein arginine N-methyltransferase 1	HUMAN	1
408	1.92	1.3	P49736|MCM2	DNA replication licensing factor MCM2	HUMAN	1
409	1.92	6.1	Q9BSH4|TACO1	Translational activator of cytochrome c oxidase 1	HUMAN	1
410	1.92	7	O95870|ABHGA	Protein ABHD16A	HUMAN	1
411	1.92	2.8	P0CW18|PRS56	Serine protease 56	HUMAN	1
412	1.92	4.9	P62701|RS4X	40S ribosomal protein S4, X isoform	HUMAN	1
413	1.9	17.5	Q56VL3|OCAD2	OCIA domain-containing protein 2	HUMAN	1
414	1.87	14.8	Q8N5K1|CISD2	CDGSH iron-sulfur domain-containing protein 2	HUMAN	2
415	1.83	3.3	Q6PI48|SYDM	Aspartate--tRNA ligase, mitochondrial	HUMAN	1
416	1.8	19.5	P12236|ADT3	ADP/ATP translocase 3	HUMAN	7
417	1.8	1.7	A0FGR8|ESYT2	Extended synaptotagmin-2	HUMAN	1
418	1.8	10.6	P18085|ARF4	ADP-ribosylation factor 4	HUMAN	2
419	1.77	1.7	Q14CZ7|FAKD3	FAST kinase domain-containing protein 3, mitochondrial	HUMAN	1
420	1.77	1.6	Q15031|SYLM	Probable leucine--tRNA ligase, mitochondrial	HUMAN	1
421	1.74	7.6	P42126|ECI1	Enoyl-CoA delta isomerase 1, mitochondrial	HUMAN	2
422	1.72	10.7	P30084|ECHM	Enoyl-CoA hydratase, mitochondrial	HUMAN	2
423	1.7	6.8	P40926|MDHM	Malate dehydrogenase, mitochondrial	HUMAN	1
424	1.7	1.3	Q12769|NU160	Nuclear pore complex protein Nup160	HUMAN	2
425	1.7	1.1	Q5JTZ9|SYAM	Alanine--tRNA ligase, mitochondrial	HUMAN	1
426	1.68	5.7	P62829|RL23	60S ribosomal protein L23	HUMAN	1
427	1.66	6.3	O75306|NDUS2	NADH dehydrogenase [ubiquinone] iron-sulfur protein 2, mitochondr	HUMAN	2
428	1.6	0.4	O60318|GANP	Germinal-center associated nuclear protein	HUMAN	1
429	1.5	2.8	Q92973|TNPO1	Transportin-1	HUMAN	1
430	1.49	2.1	O43684|BUB3	Mitotic checkpoint protein BUB3	HUMAN	1
431	1.49	2.1	Q96CU9|FXRD1	FAD-dependent oxidoreductase domain-containing protein 1	HUMAN	1
432	1.46	1.3	Q09161|NCBP1	Nuclear cap-binding protein subunit 1	HUMAN	1
433	1.44	1.3	Q8TAD4|ZNT5	Zinc transporter 5	HUMAN	1
434	1.43	3.2	Q9BZE4|NOG1	Nucleolar GTP-binding protein 1	HUMAN	1
435	1.4	9.5	P20618|PSB1	Proteasome subunit beta type-1	HUMAN	2
436	1.38	5.6	Q4J6C6|PPCEL	Prolyl endopeptidase-like	HUMAN	3
437	1.34	5.3	Q9NPL8|TIDC1	Complex I assembly factor TIMMDC1, mitochondrial	HUMAN	1
438	1.33	1.2	Q3SY69|AL1L2	Mitochondrial 10-formyltetrahydrofolate dehydrogenase	HUMAN	1
439	1.32	5	O00299|CLIC1	Chloride intracellular channel protein 1	HUMAN	1
440	1.32	6.6	O14684|PTGES	Prostaglandin E synthase	HUMAN	1
441	1.31	5.3	P62241|RS8	40S ribosomal protein S8	HUMAN	1
442	1.3	5.4	P22830|HEMH	Ferrochelatase, mitochondrial	HUMAN	1

1. Unused Score, unused protein score. For the target identification, a strict total score cut-off of 1.3 was set as the qualification criterion, which corresponded to a protein confidence interval of 95%.
2. % Cov (95), percent protein sequence coverage with the identified peptides.
